# Spatially and Chemically Resolved Visualization of Fe Incorporation into NiO Octahedra during the Oxygen Evolution Reaction

**DOI:** 10.1021/jacs.3c07158

**Published:** 2023-09-19

**Authors:** Fengli Yang, Mauricio Lopez Luna, Felix T. Haase, Daniel Escalera-López, Aram Yoon, Martina Rüscher, Clara Rettenmaier, Hyo Sang Jeon, Eduardo Ortega, Janis Timoshenko, Arno Bergmann, See Wee Chee, Beatriz Roldan Cuenya

**Affiliations:** Department of Interface Science, Fritz-Haber-Institute of the Max-Planck Society, 14195 Berlin, Germany

## Abstract

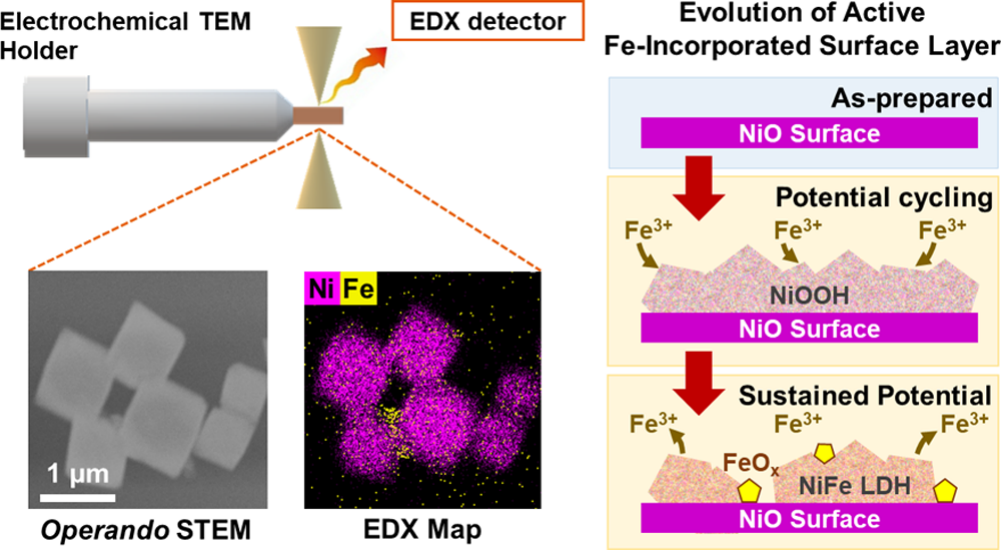

The activity of Ni (hydr)oxides for the electrochemical evolution of oxygen (OER), a key component of the overall water splitting reaction, is known to be greatly enhanced by the incorporation of Fe. However, a complete understanding of the role of cationic Fe species and the nature of the catalyst surface under reaction conditions remains unclear. Here, using a combination of electrochemical cell and conventional transmission electron microscopy, we show how the surface of NiO electrocatalysts, with initially well-defined surface facets, restructures under applied potential and forms an active NiFe layered double (oxy)hydroxide (NiFe-LDH) when Fe^3+^ ions are present in the electrolyte. Continued OER under these conditions, however, leads to the creation of additional FeO_*x*_ aggregates. Electrochemically, the NiFe-LDH formation correlates with a lower onset potential toward the OER, whereas the formation of the FeO_*x*_ aggregates is accompanied by a gradual decrease in the OER activity. Complementary insight into the catalyst near-surface composition, structure, and chemical state is further extracted using X-ray photoelectron spectroscopy, operando Raman spectroscopy, and operando X-ray absorption spectroscopy together with measurements of Fe uptake by the electrocatalysts using time-resolved inductively coupled plasma mass spectrometry. Notably, we identified that the catalytic deactivation under stationary conditions is linked to the degradation of in situ-created NiFe-LDH. These insights exemplify the complexity of the active state formation and show how its structural and morphological evolution under different applied potentials can be directly linked to the catalyst activation and degradation.

## Introduction

The electrolysis of water is a promising method to store renewable electricity generated by solar and wind power in chemical bonds (i.e., green H_2_). Nonetheless, its efficiency has been limited by the sluggish anodic half reaction, the oxygen evolution reaction (OER). For the OER in alkaline electrolytes, catalysts based on nickel oxide/hydroxides have shown excellent catalytic activity and are attractive alternatives to those operating in an acidic environment based on scarcer and more costly IrO_*x*_ or RuO_*x*_. Interestingly, Ni oxides in precleaned Fe-free electrolytes show limited activity, suggesting that the incorporation of Fe into NiO or Ni(OH)_2_ boosts their OER activity.^1−4^ This enhancement has also been reported in both Ni-based^5−8^ and Co-based electrocatalysts^6−10^ synthesized with deliberately included Fe. Notably, pure Fe oxides are poor OER catalysts, but trace amounts of Fe are already sufficient to generate highly active catalytic sites on the electrocatalysts. The formation of Fe-rich phases during reaction, on the other hand, has also been reported to cause activity degradation in mixed Ni–Fe electrocatalysts.^3,11−13^ Therefore, extensive work has aimed to understand the role of Fe in improving the performance of these catalysts. Although recent research largely points to a synergistic coupling^14^ between Ni and Fe as the reason behind the activity enhancement, different mechanisms, such as Fe^3+^ species inducing the formation of layer double hydroxide (LDH) phases in NiOOH with an increased number of active sites, the presence of high-valent metal sites like Fe^4+^ or Ni^4+^ or oxyl radicals like those found for IrO_*x*_, or a change in the intrinsic OER activity due to Fe limiting the oxidation of Ni ions during OER,^3,7,14^ have been proposed. Many studies also start from presynthesized mixed Ni–Fe structures,^15^ which makes separating the beneficial effects by the incorporated Fe from any morphological changes in the metal oxide host highly challenging. Therefore, the key to rationalizing the catalytic impact of Fe lies in elucidating how the near-surface structure and composition of these catalysts change under reaction conditions as a function of the Fe added.

Obtaining reliable insight into a working catalyst’s surface is, however, a nontrivial challenge. Most prior studies relied on operando spectroscopy^16^ to track the changes in the catalyst structure, metal oxidation state,^17,18^ or metal–ligand charge redistribution.^19,20^ Unfortunately, these ensemble-averaging methods cannot resolve the local chemical and structural changes induced on a catalyst surface by trace metal species at high spatial resolution. Conversely, precise knowledge regarding the nature of the catalyst surface, the location of Fe incorporations, and the exact Fe species that is beneficial for electrocatalytic performance is required for building realistic surface models for theoretical studies. There are very few studies that describe how the surface morphology of Ni (hydr)oxide electrocatalysts is altered during reaction by the addition of Fe, especially at the low Fe loadings in the early stages of incorporation, where the activity changes are most drastic. Previously, Deng et al.^21^ showed with in situ electrochemical atomic force microscopy (AFM) that the incorporation of Fe into single-layered Ni(OH)_2_ sheets at high anodic potential can cause significant structural changes, which include volume expansion of the nanosheets and a transformation into loosely packed nanoparticles. The prevailing hypothesis based on these scanning probe microscopy studies is that Fe is first added to the catalyst corners and edges,^22^ but these measurements lack chemical sensitivity and so, they do not provide direct evidence regarding the Fe distribution on the catalyst under electrochemical conditions and how it can be related to the morphological changes seen in the underlying Ni (hydr)oxide host.

Here, we first tracked the evolution of octahedral NiO catalyst particles with flat surface facets in Fe-containing alkaline electrolytes^23,24^ during OER using operando electrochemical (scanning) transmission electron microscopy (EC-(S)TEM)^25^ coupled with energy dispersive X-ray spectroscopy (EDX) mapping^26^ to reveal their morphological and chemical changes under applied potential and in the presence of Fe. STEM-EDX can provide us with spatially resolved maps that follow multiple elemental signatures concurrently, but so far, it has been under-utilized in time-resolved liquid phase TEM studies. With EC-TEM, we were able to image these particles in situ inside the electrolyte and under applied potential with a few nanometer resolution,^27^ while the EDX maps allow us to follow the catalyst composition at different reaction times. Thanks to the well-defined surfaces of these NiO octahedra, we were able to visualize how the electrocatalyst morphology and near-surface composition changed during the incorporation of electrolyte Fe species. We then selected key points of the reaction as indicated by these operando studies for detailed ex situ investigations to obtain more insight into the surface transformations and to relate these transformations to the changes in the electrochemical and catalytic properties. These studies reveal that the catalyst surface restructures in response to different applied potentials, which can in turn create or destroy active NiFe layered double hydroxide (NiFe-LDH) structures on the NiO surface. These observations are further supported by complementary studies using other operando and time-resolved techniques such as X-ray absorption spectroscopy (XAS) and Raman spectroscopy.

## Results and Discussion

**Figure 1:**
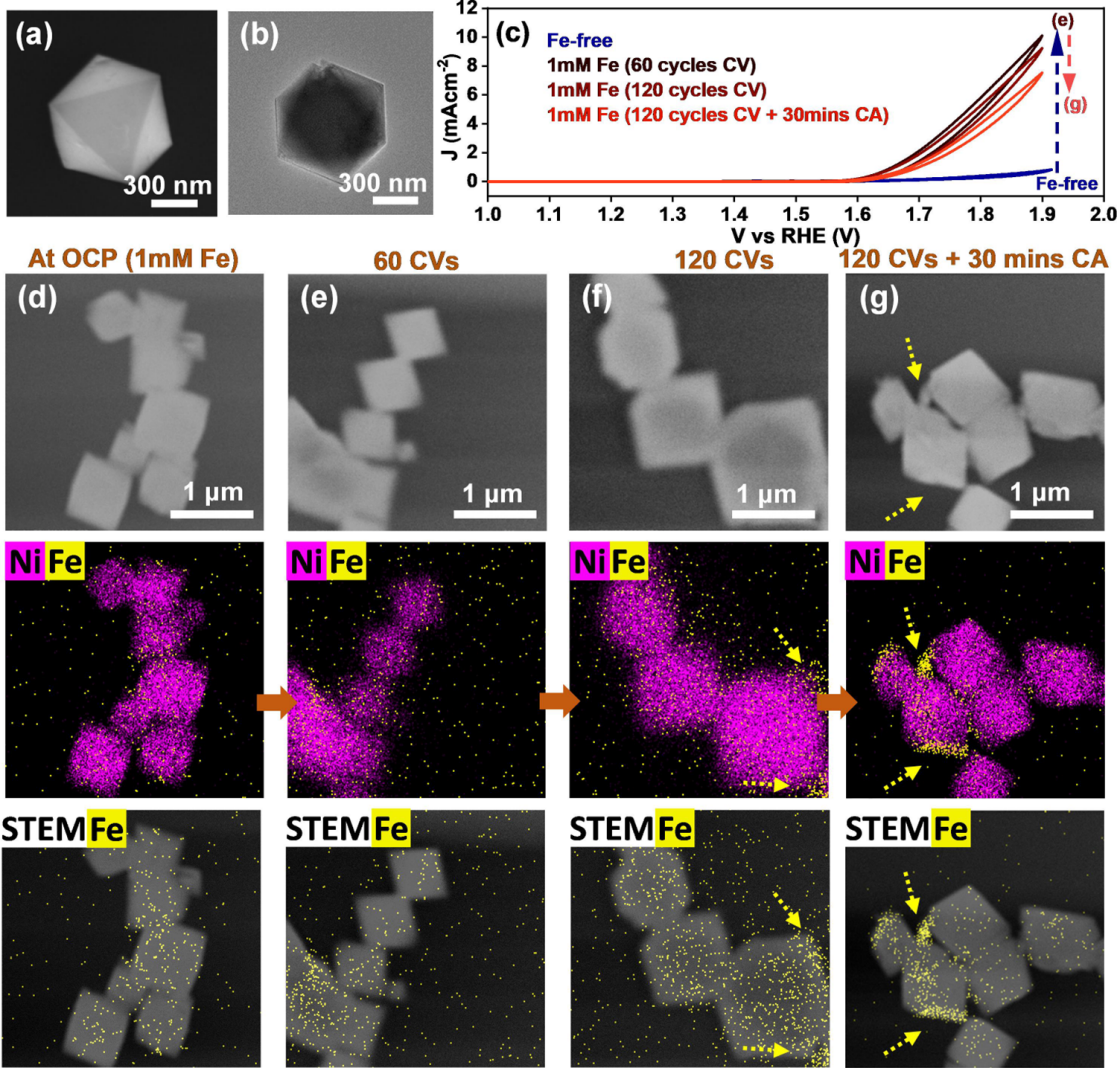
(a) Ex situ scanning electron (SEM) and (b) transmission electron (TEM) microscopy images of a NiO octahedron. (c) Cyclic voltammograms compare the activity after 10 cycles OER operation in a borate buffer without Fe to that obtained after different reaction times with 1 mM Fe(NO_3_)_3_ added into the electrolyte. (d–g) In situ STEM images, combined Ni/Fe chemical EDX maps, and STEM/Fe EDX maps of NiO octahedra acquired in a 0.5 M borate buffer +1 mM Fe(NO_3_)_3_ solution: (d) at OCP, (e) after 60 cycles of CV, and (f) after 120 cycles of CV from 0.7 to 1.9 V_RHE_. (g) STEM image and Fe map collected after an additional 30 min at 1.7 V_RHE_ after 120 CV cycles. Aggregated Fe is highlighted in (f) and (g) with dashed arrows. The EDX maps in (e,f) are collected during CV, and the EDX map in (g) is collected under an applied potential at 1.7 V_RHE_.

A SEM and TEM image of an as-synthesized NiO octahedron is shown in Figure 1a,b, respectively. Additional ex situ TEM images and STEM-EDX maps of these NiO octahedra are presented in Figure S1. XPS and ICP-MS measurements of the as-prepared samples only present signatures of Ni and exclude the presence of Fe (≤0.03 mol % from ICP-MS, Table S1). X-ray diffraction confirms that the octahedra are crystalline cubic nickel oxides (Figure S2, space group *Fm*3̅*m*).

Figure 1c–g shows the operando EC-TEM results that were acquired while 0.5 M borate buffer flowed continuously through the liquid cell. The electrochemical cycling experiments (Figure S3) were carried out initially in the absence of Fe in the electrolyte. After 10 cycles of cyclic voltammetry (CV) from 0.7 to 1.9 V_RHE_, the injected electrolyte was changed to 0.5 M borate buffer, with added 1 mM Fe(NO_3_)_3_. A short movie acquired during the switch between the Fe-free and Fe-containing electrolyte is provided as Movie S1. Figures 1d–g and S4 show the STEM images and Fe maps that were collected in situ during the OER before and after the addition of Fe into the electrolyte. Figure 1c shows representative CVs collected during the experiment. To avoid beam-induced artifacts caused by the extended acquisition times required to obtain reasonable EDX maps, we looked at new octahedral particles at each point of time. These in situ EDX maps will, nonetheless, be inherently noisy due to the low electron beam currents used during these experiments and the short acquisition times imposed by the need to avoid averaging over multiple potential cycles. We also mention here that due to the Ni X-rays having sufficient energy to excite secondary fluorescence of Fe X-rays, there is a very weak Fe signal in the EDX spectra acquired from the as-synthesized samples. This is an artifact that originates from Fe present in the TEM column itself.

During cycling in a 0.5 M borate buffer, a Ni^2+^/Ni^3+^ redox transition was observed at 1.46 V_RHE_ from the CV (Figure S5), but no significant shape changes due to the oxidation of NiO to NiOOH were seen in the in situ STEM images (Figure S6). Figure 1d shows the in situ image and map acquired at open circuit potential (OCP) in 0.5 M borate buffer +1 mM Fe(NO_3_)_3_ electrolyte, which indicated that Fe was not present in amounts detectable by in situ EDX mapping in the octahedra when Fe was first introduced into the electrolyte and before the potential was applied. After applying potential cycles in Fe-containing electrolyte, the OER potential of the sample at 1 mA cm^–2^ began to shift toward lower potentials (from 1.90 to 1.65 V_RHE_) after 60 cycles as shown in Figure 1c where the next EDX map is collected. Again, the incorporation of Fe into the octahedra is not obvious in the first EDX map collected in the Fe-containing electrolyte after 60 cycles (Figure 1e).

After 120 cycles, a faint Fe-containing surface layer and diffuse Fe aggregates can now be seen in the EDX map (Figure 1f), with a small but clear Fe peak visible in the integrated EDX spectrum (Figure S7). The acquired CV indicates a slight drop in the anodic current (Figure 1c). Next, we performed chronoamperometry (CA) at a constant potential of 1.7 V_RHE_ for another 30 min (Figure S8) to understand how an extended reaction time impacts the surface layer with Fe incorporated. Interestingly, the EDX maps acquired after 30 min of CA (Figure 1g) indicate that there was further aggregation on the surface of the catalysts. Moreover, we can infer that the Fe content was changing in the Fe-containing layer, despite the poor signal-to-noise ratios, by comparing the EDX maps collected during CV and CA. As shown in the bottom row of Figure 1d–g, where we overlaid the STEM images and their corresponding Fe maps, the surface-incorporated Fe increased during the applied CVs but decreased during subsequent CA operation. During CA, the current decreased gradually over 30 min (Figure S8). A CV collected after 30 min also confirmed the drop in current (Figure 1c).

It should be noted that these shifts were not seen in the samples reacted in Fe-free borate buffer during the first 120 cycles. As depicted in Figure S9, ex situ benchtop measurements using identical octahedral particles dropcasted on a EC-TEM carbon chip only showed ∼2× improvement of the OER activity in Fe-free electrolyte as a function of the reaction time, which is a result of the activation of the catalyst due to reaction-induced structural modifications.^23^ On the other hand, a ∼ 10× improvement in the activity (current density) was measured for similarly prepared samples in the Fe-containing 0.5 M borate buffer (Figure S5). Thus, even though a small improvement in the activity is expected also in the latter sample due to operando surface restructuring even in the absence of Fe, the improvement in the electrocatalytic performance is much more significant when Fe impurities are introduced. The same NiO catalysts dropcasted on carbon paper and reacted in the Fe-containing electrolyte also showed a shift of the OER onset potential toward lower potentials at 1 mA cm^–2^ (Figure S10, from 1.82 to 1.65 V_RHE_). Thus, the electrochemical behavior of the NiO octahedra used in our EC-TEM experiments is consistent with that described in previous work where Fe was deliberated introduced into the electrolyte.^28^

**Figure 2:**
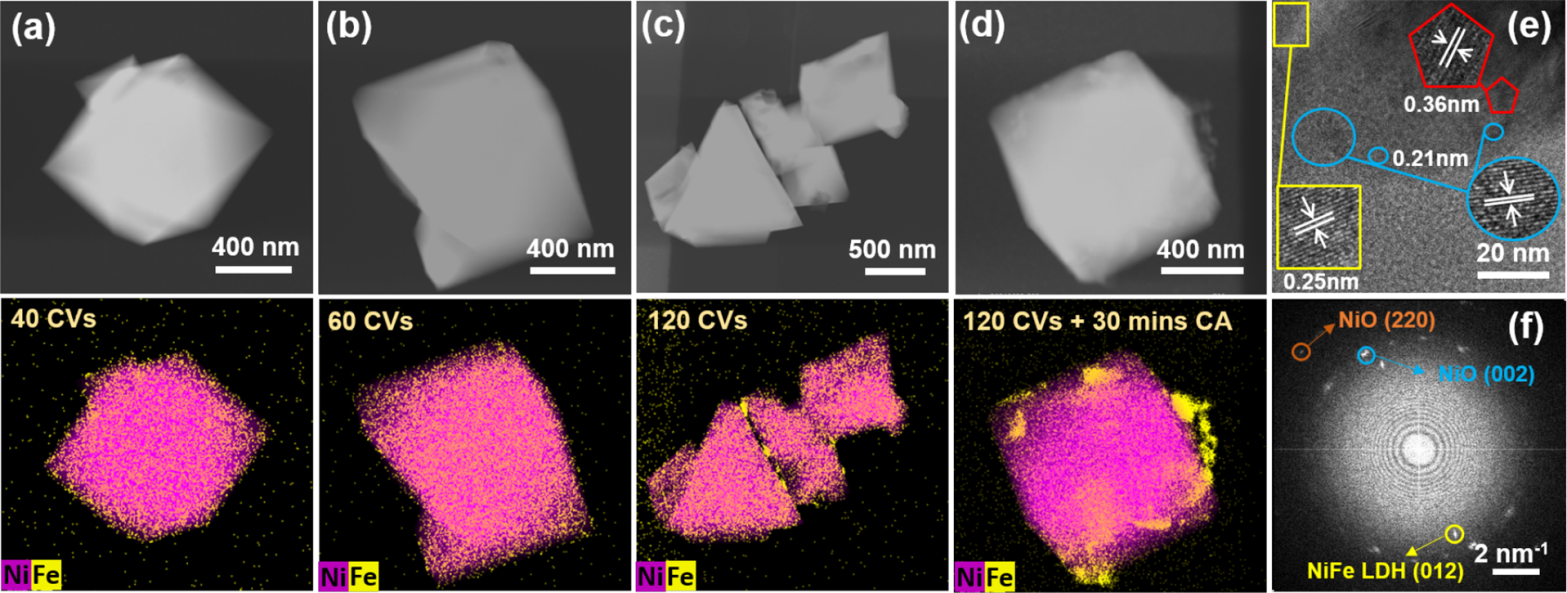
Ex situ EDX maps of NiO catalysts after in situ EDX experiments in 0.5 M borate buffer +1 mM Fe(NO_3_)_3_ solution under different reaction conditions: (a) after 40 cycles of CV scanning, (b) after 60 cycles of CV scanning, (c) after 120 cycles of CV scanning, and (d) after 120 cycles of CV scanning and CA measurements for 30 min at 1.7 V_RHE_. (e,f) HRTEM and fast-Fourier-transform (FFT) images of NiO after 120 cycles of CV scanning and CA measurements for 30 min at 1.7 V_RHE_. The lattice spacing was evaluated using both FFT image and HRTEM image. We used at least 10 parallel planes in HRTEM image.

Samples were also extracted from our operando EC-TEM experiments after different OER times, and the changes in their surface structures that occurred during reaction were examined using extended ex situ STEM-EDX mapping with better signal-to-noise ratios and high-resolution TEM (HRTEM) analysis (Figure 2). Figure 2a*–*c shows the EDX maps of NiO catalysts after 40, 60, and 120 CV cycles in the Fe-containing electrolyte, respectively. Images and maps of additional particles can be found in Figures S11 and S12. These ex situ maps support our in situ observations showing that Fe was incorporated initially into NiO as a thin surface layer during the OER in the 1 mM Fe-containing electrolyte, and with extended reaction time (120 cycles), small Fe-containing aggregates began to form on the electrocatalyst surface (Figure 2c). After the subsequent 30 min of sustained CA, the Fe aggregates became larger (Figure 2d). We further analyzed the EDX maps (Figure 2a–d) by extracting line profiles and quantified the Fe-to-Ni ratios across the octahedra (Figure S13). After CV, we found a Fe-to-Ni ratio of 20–30% Fe on the edge of the octahedra, and this ratio decreased systematically to ∼10% toward the octahedra’s center, which agrees with a sample geometry where only the surface is enriched with Fe. The EDX line profiles also indicate a decrease in the surface Fe content to ∼15% after applying CA for 30 min (Figure S14). We emphasize here that the analogous results from the in situ experiments shown earlier indicate that the Fe aggregates are not byproducts of the electrolyte drying.

Selected area diffraction (SAED) patterns taken from the catalysts before (Figure S15a) and after (Figure S15b) the OER both show strong (220) diffraction spots for NiO and confirm that the Fe incorporation only altered the surface structure of the octahedra. Lattice fringes found in HRTEM images of the octahedra and their corresponding fast Fourier transform (FFT) indicate interplanar spacings of 0.21 nm in the thicker regions of the sample, which correspond to the (002) planes of NiO, while certain areas near the octahedra edges show fringe spacings of 0.25 nm (Figures 2e,f and S16). The latter spacing can be associated with the spacing of the (012) lattice plane of NiFe-LDH^29^ or the (311) lattice plane of NiFe_2_O_4_.^29^ To enhance the visualization of this new structure, we extracted filtered real-space images of the areas that present a 0.25 nm lattice fringe, which appear as small domains on the octahedron surface (Figure S17). HRTEM images of the Fe aggregates, on the other hand, show lattice spacings of 0.36 nm, which can be assigned to the (012) plane of hematite α-Fe_2_O_3_,^30^ in agreement with previous work performed under high anodic potentials (>1.4 V_RHE_).^31,32^

**Figure 3:**
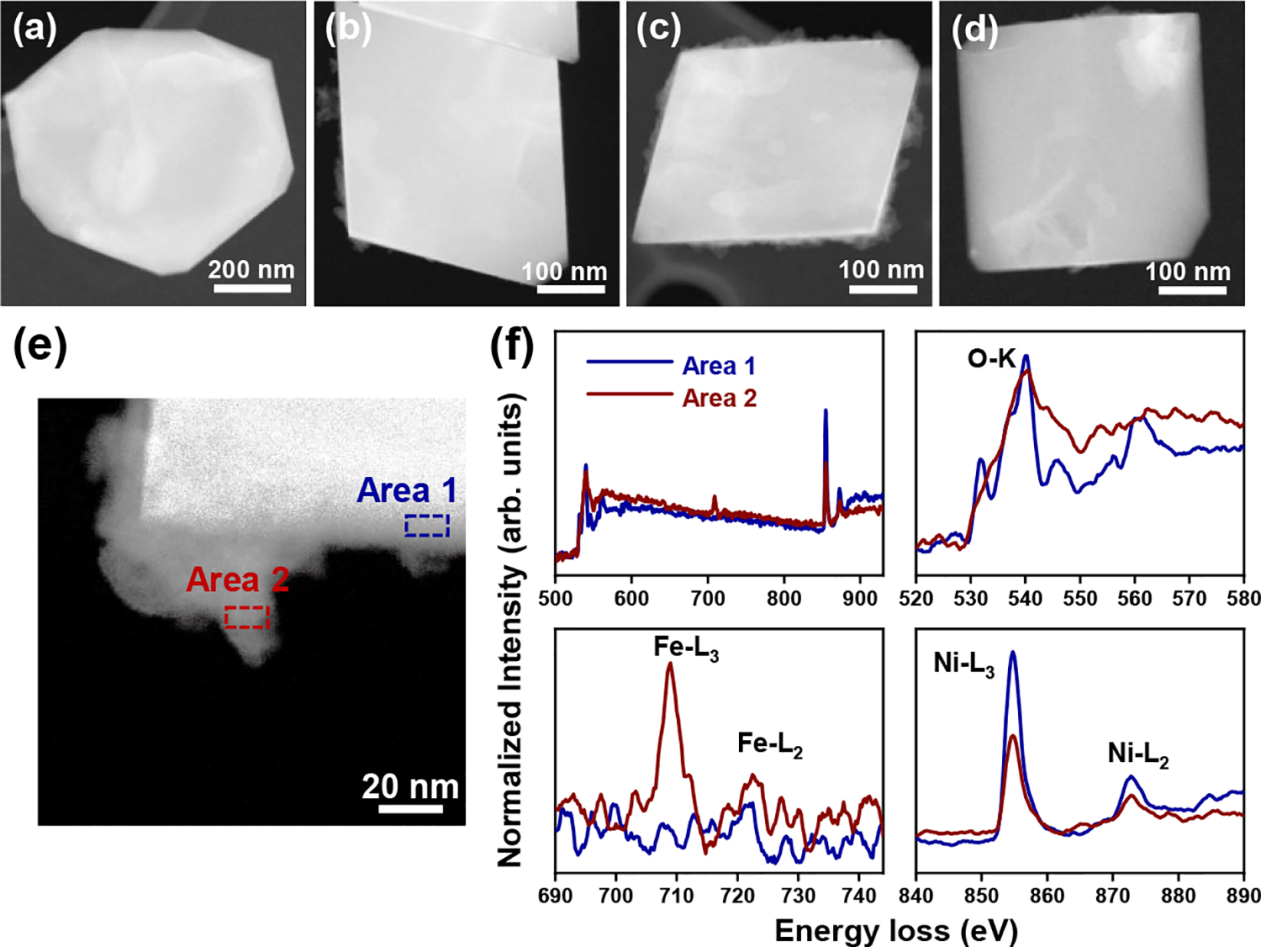
Ex situ annular dark-field images of NiO catalysts: (a) as-prepared, (b) after 60 cycles of CV scanning, (c) after 120 cycles of CV scanning, and (d) after 120 cycles of CV scanning and CA for 1 h at 1.7 V_RHE_ in 0.5 M borate buffer +1 mM Fe(NO_3_)_3_ solution using our standard benchtop electrochemistry setup. (e) Annular dark-field image of NiO after 60 cycles of CV scanning in 0.5 M borate buffer +1 mM Fe(NO_3_)_3_ solution. (f) Background-subtracted EELS spectra and O, Fe, and Ni edges extracted from the spectra collected at the positions marked with red and blue rectangles in (e). The samples were dropcasted on a glassy carbon support and then transferred onto a standard TEM grid after reaction.

To visualize these catalyst changes at higher spatial resolution, we performed ex situ aberration corrected STEM on octahedra that were reacted on a bulk glassy carbon plate in Fe-containing electrolyte and then transferred onto a TEM grid (see Supporting Information, Note 1 for a description of the control experiments we performed to confirm the consistency between our EC-TEM studies and benchtop experiments). The acquired annular dark-field STEM images (Figure 3b,c) indicate that there were faint flakelike features on the NiO octahedra that were tens of nanometers in size after 60 and 120 cycles of CV. Due to their weak contrast, these flakes were not visible in the in situ STEM images. EELS spectra (Figures S18 and S19) were collected from the sample octahedra after 60 CV cycles and show that the surface flakes contain both Fe and Ni. Figure 3e,f compares two measurements extracted from Figure S18, one from the flake and the other from the NiO surface. The O, Fe, and Ni edges after background subtraction are shown in Figure 3f. First, the features of the O edges are consistent with O being in the form of lattice O in the NiO and being in a more disordered state in the flake.^33^ Second, the Fe edge data confirm that Fe was localized at the flakes, but the data that could be obtained were too noisy to allow for quantification of the Fe oxidation state.^34^ Third, a comparison of the L_3_ to L_2_ ratios in the Ni edges^35^ indicate that Ni in the flakes has a higher oxidation state as compared to Ni at the octahedron surface, suggesting that the flakes are the result of NiO being oxidized to oxyhydroxide as was reflected in the CVs. After sustained CA of 1 h, the density of these flakes decreased significantly (Figure 3d). Surface flakes were also seen in the sample in Fe-free electrolyte after 120 CVs, and they were also removed after stationary OER (Figure S20), confirming that they were a result of NiO surface restructuring. Additional SEM and STEM images of these samples are provided in Figures S21 and S22.

Taken together, our microscopic observations indicate that the improvement in the catalytic properties of the octahedra under the OER conditions is associated with Fe being incorporated at the surface as the NiO surface restructures under applied potential cycling. Our results further imply that the amount of Fe that can be accommodated within this superficial layer is limited and further Fe incorporation is impeded once the solubility limit of Fe in the Ni (hydr)oxide host is reached. Adding more Fe instead leads to Fe segregation in the form of FeO_*x*_ aggregates, which in turn causes the observed decrease in the measured OER current. These in situ-generated flakes are also found to degrade at sustained anodic potentials, which associates the degradation with the decrease in catalytic performance over time during sustained OER and explains the decrease in uniformly distributed surface Fe and the added FeO_*x*_ formation, as shown by the EDX maps.

**Figure 4:**
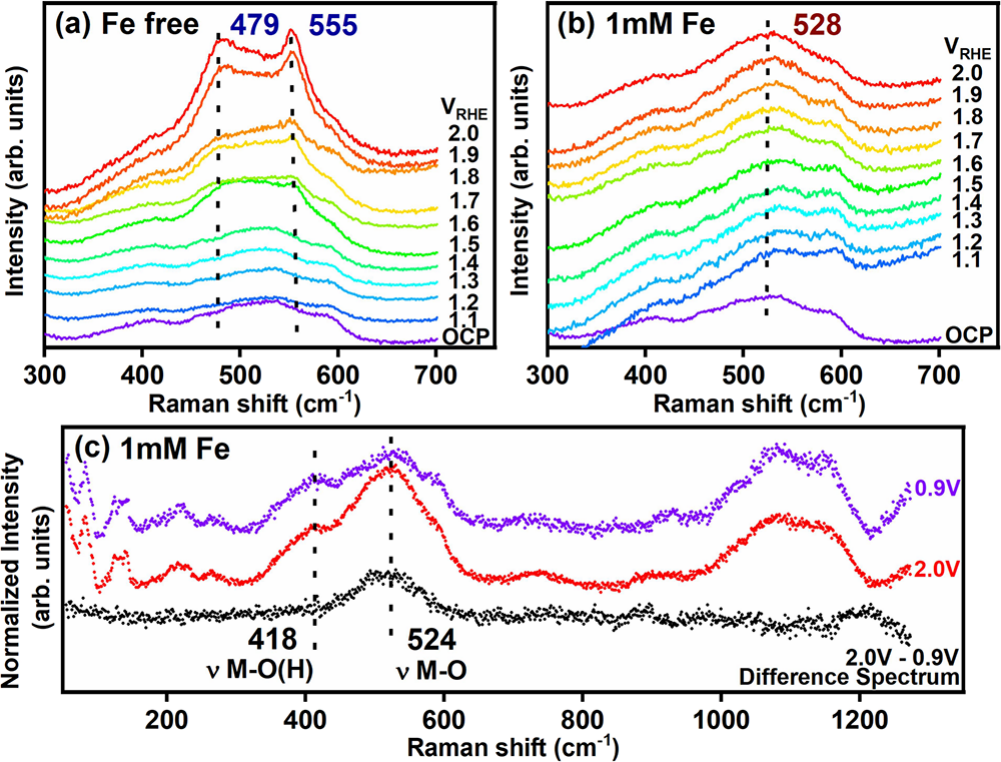
Operando Raman spectra collected in the potential range from 1.0 to 2.0 V_RHE_ (a) in 0.5 M borate buffer solution and (b) in 0.5 M borate buffer +1 mM Fe(NO_3_)_3_ solution. The measurements were performed under a constant applied potential. (c) Operando Raman spectra normalized based on the electrolyte band at 83 cm^–1^. Baseline subtraction was performed by fitting the function to a polynomial baseline with order 4 and 1.5 noise tolerance using WIRE 5.2 from Renishaw. Black spectra are the result of the subtraction of the spectra at 0.9 V _RHE_ (blue) from the one at 2.0 V_RHE_ (red) in 0.5 M borate buffer +1 mM Fe(NO_3_)_3_ solution.

To achieve better understanding into these morphological and chemical changes, we performed complementary operando Raman spectroscopy, operando XAS, ex situ X-ray photoemission spectroscopy (XPS), and time-resolved ICP-MS studies. Figure 4a,b displays operando Raman spectra that were acquired under the OER conditions in a Fe-free and Fe-containing (1 mM Fe(NO_3_)_3_) electrolyte, respectively (full-spectrum data for the Fe-free and Fe-containing electrolyte are provided as Figure S23a and S23b, and the electrochemical data acquired during the experiments are shown in Figure S24). At OCP, the Raman spectrum acquired in both the Fe-free and the Fe-containing electrolyte possesses several modes centered at 409, 518, 900, and 1090 cm^–1^ respectively, which are in good agreement with the reported values of NiO.^36,37^ When the anodic potentials were applied in the Fe-free electrolyte, additional weak bands emerged at 479 and 555 cm^–1^ (Figure 4a), which match the E_g_ bending vibration and the polarized A_1g_ stretching mode of Ni–O(H) in NiOOH, respectively.^23,38^ The oxidation of Ni from a 2+ to a 3+ oxidation state is consistent with the redox transition at ∼1.5 V_RHE_ (Figure S24a). Above ∼1.5 V_RHE_, the behavior in the Fe-containing electrolyte started to deviate from the Fe-free case where a new broad peak at 528 cm^–1^ emerged (Figure 4b). The peak around 528 cm^–1^ has been as interpreted thin, disordered NiFe-LDH^39,40^ or as FeOOH nanoclusters supported on NiOOH.^41^ We assign it to the former due to its agreement with the TEM data and the absence of NiOOH bands in the spectra. The lack of the characteristic NiOOH bands in the Raman spectra collected in the Fe-containing electrolyte is also explained by the Fe incorporation into the NiOOH structure resulting in a large degree of disorder^3,23^ as compared to Figure 4a. To clarify the features of the broad peak, the Raman spectra are normalized based on the band at 83 cm^–1^ that is due to the borate buffer electrolyte^42^ (Figure S26) and the spectrum acquired at 0.9 V_RHE_ is subtracted from the spectrum acquired at 2.0 V_RHE_. A wide band centered at 524 cm^–1^ and a weak shoulder at 418 cm^–1^ are clearly seen in the difference spectrum (Figure 4c). Similar spectral features appear at and above 1.8 V_RHE_ (Figure S27), which is lower than the upper bound of our CV protocol but close to the potential applied during CA, implying that surface restructuring is associated with higher anodic potentials. We also confirmed the formation of Fe oxides (aggregates observed locally via TEM/EDX), specifically hematite α-Fe_2_O_3_, after our extended electrochemistry experiments using ex situ Raman measurements (Figure S28).

We further examined ex situ reacted octahedra using X-ray photoelectron spectroscopy (XPS) to obtain surface-sensitive composition information about near-surface Fe incorporation prior to the formation of FeO_*x*_ surface aggregates. A Ni:Fe atomic ratio of ∼2.7:1 was determined for the NiFe-LDH formed after 60 min OER in Fe-containing electrolyte from fitting the Ni-2p and Fe-2p spectra (Figure S29). This Ni:Fe ratio agrees with our EDX results and is, interestingly, consistent with previous operando XAS studies of Ni–Fe thin films during OER,^11^ which reported a maximum activity at 20–30% Fe, and its continuous drop as the Fe content was further increased.

**Figure 5:**
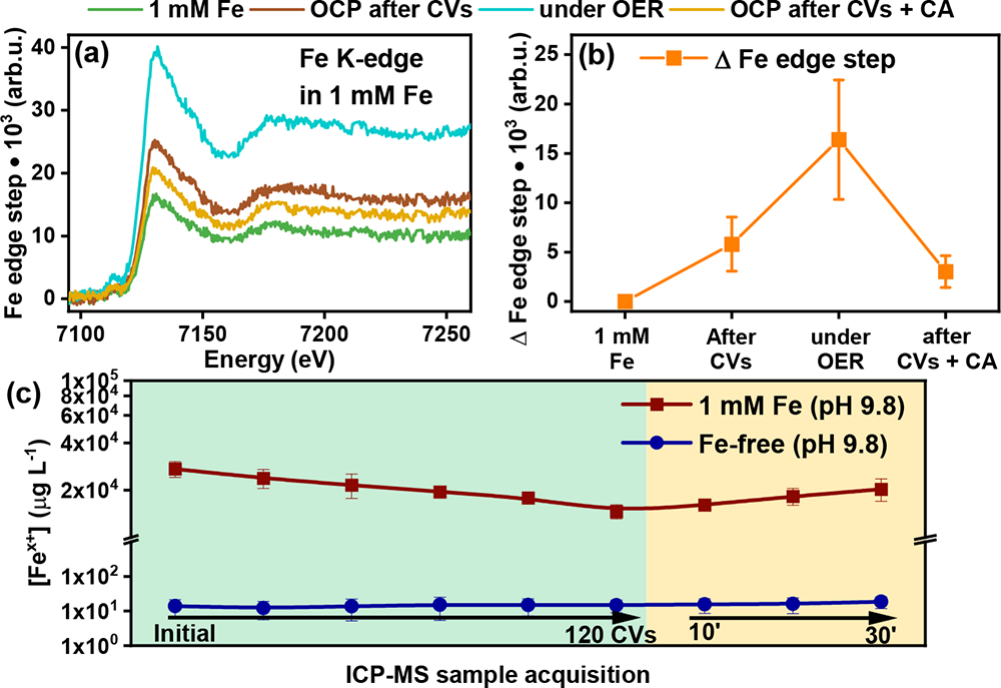
(a) Fe K-edge XANES data measured in 0.5 M borate buffer +1 mM Fe(NO_3_)_3_ solution and (b) corresponding increase in the Fe edge step intensity under reaction conditions. The depicted Fe edge step values were obtained by averaging results from three Fe K-edge XANES scans, and the standard deviations of the obtained values are used as uncertainties. (c) ICP-MS results of Fe element in the electrolyte. Quantification of Fe uptake/loss in NiO octahedra was performed by replicating the EC-TEM experiments in an H-cell configuration. The electrolyte samples were collected during cycling (0, 10, 20, 40, 60, and 120 CVs) and CA (1 mL every 10 min) from the WE compartment.

To track the uptake of Fe from the electrolyte during the OER, we carried out operando X-ray absorption spectroscopy (XAS) measurements at the Ni and Fe K-edge in Fe-free and 1 mM Fe-containing electrolytes and time-resolved ICP-MS measurements (shown as Figure 5a–c, respectively). The XAS samples were measured: (1) dried in their as-prepared state, (2) in 1 mM Fe-containing or Fe-free electrolyte at the OCP after 60 CV cycles (0.8–2.0 V_RHE_, 20 mV·s^–1^), (3) under the OER conditions at a constant applied potential of 1.6 V_RHE,_ and (4) at the OCP again after one additional hour of CA at 1.6 V_RHE_. All of the Ni K-edge X-ray absorption near-edge structure (XANES) profiles in the as-prepared state and under working conditions are very similar to the spectra of a NiO reference (Figure S31) although there is a slight difference in the intensity and position of the white line at ∼8351 eV. Linear combination fitting (Figure S32, LCF) indicates that the as-prepared samples are mainly NiO (95%), with small amounts of Ni(OH)_2_ (5%).

In general, the XANES spectra at the Ni K-edge of our catalyst in both Fe-free and 1 mM Fe indicates that most of the Ni species are in the 2+ state, regardless of electrochemical conditions or the presence/absence of Fe species. This indicates that the oxidation of Ni is only limited to the surface of our particles, which we cannot resolve in our sample-averaged data, as expected given the significant bulk phase contribution in these relatively large particles. Similarly, fitting of Fourier-transformed extended X-ray absorption fine structure (FT-EXAFS) data for the first two coordination shells (Figure S31c,d) suggests similar Ni–O and Ni–metal coordination numbers for samples under OER in Fe-free and 1 mM Fe containing electrolytes that matches well the values for the bulk NiO reference. The small differences in Ni–O distances observed in the measurements with and without Fe in the electrolyte do not exceed the uncertainties in the EXAFS analysis. EXAFS fitting results are provided in more detail in the Supporting Information Table S2.

The operando XANES profiles at the Fe K-edge, on the other hand, show intriguing features during the reaction. The shape and position of the main features in non-normalized Fe–K edge XANES spectra did not vary significantly under the different electrochemical conditions (Figure 5a) and so, the average oxidation state of Fe remained 3+ (Table S3). Although the shape of Fe K-edge XANES profiles agrees with a α-Fe_2_O_3_ reference spectrum (Figure S36), we cannot differentiate between Fe existing in the form of an LDH or as Fe oxide aggregates from these measurements. Conversely, we found that the absorption jump at the Fe K-edge edge changed under different conditions. From Figure 5b, we can see that the intensity of the Fe edge step increased after 60 CV cycles, consistent with the Fe incorporation into the NiO catalyst as seen in the EC-TEM results, and increases further (∼2×) under stationary OER conditions. More interestingly, the step edge height decreased roughly back to the level measured after 60 CV cycles when the samples were returned to the OCP after 1 h of CA, which ruled out beam-induced deposition of Fe being the cause of the step intensity increase (discussed further in Supporting Information Note 2). While the Fe K-edge is sensitive to the Fe concentration variations in both, the electrocatalyst and the electrolyte near the electrode surface (about 200 μm probing depth), our time-resolved STEM-EDX results indicate that Fe did not incorporate deeper into the bulk of these octahedral NiO particles over time and only formed a superficial NiFe-LDH layer. Therefore, the reversible change in the Fe K-edge XANES spectra suggests an accumulation of Fe from the electrolyte at the electrode surface under applied positive potential. In this regard, we reiterate that we did not identify changes in the Fe chemical state or coordination during OER, suggesting that Fe^3+^ is the primary species present during OER, which is in accord with previous work discussing an associated decrease in the overpotential.^43−45^

As mentioned earlier, our results agree with previous operando XAS studies of codeposited Ni–Fe films by Friebel et al.^11^ that reported a transition from a mixed NiFe oxyhydroxides surface to one covered by inactive FeOOH with increasing Fe content. It should be highlighted that analogous results are obtained despite the different methods used in both studies for the addition of Fe. Furthermore, our operando XANES and ex situ EELS data complemented each other by showing respectively the changes in the Ni and the Fe components and confirmed that Fe is incorporated in NiOOH, which is also consistent with the study by Friebel et al.^11^ We similarly observed that Fe is present in the 3+ state although in our case, the Fe edge intensity consisted not only of Fe incorporated into the Ni–Fe LDH but also an additional contribution, possibly Fe from the electrolyte, as indicated by the edge jump.

To clarify the reason behind the edge jump, we performed time-resolved ICP-MS experiments, which revealed an initial Fe uptake from the electrolyte and its subsequent partial release during extended operation (Figure 5c). In particular, we observed a continuous drop in the electrolyte’s Fe concentration during cycling in the Fe-containing electrolyte. A subsequent switch in the electrochemical measurement protocol to sustained CA, conversely, led to a gradual increase in the Fe concentration, but to a maximum level that was still significantly lower than the initial Fe concentration in the electrolyte. It indicates that some Fe remains on the NiO octahedra surface but more likely as FeO_*x*_ aggregates rather than NiFe-LDH based on the TEM data. The Ni concentrations in the electrolyte were at least 2–3 orders of magnitude lower than the Fe concentrations and remained largely constant during both CVs and CA in both Fe-containing and Fe-free electrolytes although the Ni concentration appeared to increase slightly over time during CA in Fe-containing electrolyte as shown in Figure S40. The minimal Ni leaching during operation in these measurements are in line with the high stabilities found during previous ICP-MS measurements,^46^ while the gradual increase in Ni concentration in Fe-containing electrolyte during CA can be explained by some Ni leaching into electrolyte as the LDH degraded. The details of these experiments are further described in the Supporting Information Note 3. We can further quantify the Fe uptake by comparing it against the total Ni content in catalysts loaded and estimate the Ni:Fe molar ratio during the OER (calculations described in Note 3). The calculations show that the Ni:Fe ratio increased from ∼1:1 at 10 CV cycles to ∼1:4 at 120 CV cycles and decreased to ∼1:2 after 1 h of CA. These ratios indicate significantly higher Fe content than that expected from 3:1 Ni:Fe ratio we obtained for the thin LDH surfaces from ex situ XPS and EDX measurements, especially considering that the calculated ratios from the ICP-MS measurements include all the Ni atoms present in the catalysts. Therefore, these results also imply that a significant amount of aqueous Fe species was bound to the working electrode under the OER conditions as suggested by the higher edge intensity found in the XAS measurements (Figure 5b). Recently, it was shown that there is dynamic exchange between Fe species in the electrolyte and Fe located on a transition metal (oxy)hydroxide host during OER in Fe-containing electrolytes.^46^ Hence, we speculate that the Fe edge jump observed in the XAS measurements and enhanced surface Fe uptake found in the ICP-MS measurements are the result of an increased adsorption of Fe species from the electrolyte on the roughened catalyst surface created by restructuring under applied positive potential.

These studies starting from morphologically and structurally well-defined precatalysts and using a unique combination of time-resolved operando microscopy and spectroscopy methods supported by more conventional analysis provide a consistent picture for the incorporation of Fe into Ni oxides/hydroxides during the OER and the subsequent degradation of these Fe-enriched features during sustained reaction. First, we identify an incremental restructuring of the pristine NiO catalyst surface during cycling, which in the presence of Fe in the electrolyte forms a superficial NiFe-LDH layer. This transformation is associated with a beneficial shift in the onset potential for OER toward lower values. A saturation of Fe incorporated into NiOOH subsequentially leads to Fe_2_O_3_ precipitation, which slowly deactivates the electrocatalyst. The NiFe-LDH created in situ also degrades during sustained OER, leading to less LDH on the catalyst surface over time, more FeO_*x*_ aggregates, and a further drop in the electrocatalyst performance. This loss of the active surface structures is reflected in the decrease in the operando XAS Fe edge intensity, reduced surface Fe in the STEM-EDX maps, and more Fe detected in the electrolyte by the ICP-MS measurements. This process, however, does not return the NiO back to an entirely pristine state, as indicated by the still increased Fe K-edge XAS signal measured after reaction (Figure 5b) in comparison to that recorded prior to any electrochemical treatment, which we attribute mainly to the residual FeO_*x*_ surface aggregates.

In short, our studies indicate that the NiO surface does not just form NiFe-LDH structures during the OER in Fe-containing electrolyte. These active structures can also be destroyed even while Fe remains present in the electrolyte under an applied static potential. In a way, these results parallel the stabilization of active Fe sites via dissolution and redeposition reported previously,^46^ while demonstrating how these processes may be associated with changes in the metal oxide surface. Furthermore, our work showing the complex restructuring of a highly crystalline and often assumed stable NiO surface under reaction conditions implies that the true active structures cannot be easily determined without prior knowledge of the catalyst state from operando experiments. It further suggests that limited fundamental understanding can be obtained from theoretical calculations that use lattice structures derived from the starting nickel (hydr)oxide or NiFe-LDH precatalysts or their terminal states after reaction and more efforts needs to be applied toward developing models that can account for an evolving catalyst surface. Finally, we have demonstrated how EC-TEM with chemical mapping and complementary operando spectroscopy can be synergistically combined to advance our understanding of electrocatalyst transformation under reaction conditions. Our study also features how to use these results to inform key reaction times that should be selected for in-depth study using complementary techniques in order to probe the different lengths and time scales that are relevant for catalysis.

## Conclusions

In summary, octahedral NiO particles were used as model OER precatalysts and investigated under operando reaction conditions via EC-TEM (with STEM-EDX mapping), Raman spectroscopy, and XAS to probe the incorporation of Fe impurities during the OER. We were able to visualize in situ how minute amounts of Fe present in the electrolyte change the surface composition of the NiO octahedra when we applied anodic potentials. We also revealed that during potential cycling in Fe-containing electrolyte, the NiO surface first transforms into a superficial layer of NiFe-LDH. Then, for longer cycles, additional Fe deposits on the catalyst surface as an aggregated FeO_*x*_ phase when Fe can no longer be accommodated in the LDH layer. During the OER at a constant applied anodic potential, this LDH layer degrades, resulting in extended FeO_*x*_ aggregation and some Fe returning to the electrolyte. The initial formation of the NiFe-LDH leads to the shift toward a lower OER onset potential, while the subsequent formation of FeO_*x*_ aggregates reduces the accessible Ni-rich active surface area by poisoning the surface. The degradation of the in situ-created NiFe-LDH structures during sustained operation results in further loss of catalytic performance. These results illustrate how Fe species alter the surface of nickel-based catalysts and provide insight into how the restructuring of a working catalyst in response to applied potentials results in changes to the active surface. Our research also demonstrates how spatially and temporally resolved EC-TEM studies can unveil the chemical changes that take place in electrocatalysts under reaction conditions and how these operando experiments can inform subsequent detailed investigations that reveal the actual catalyst surface during reaction.

## Supporting Information Available

Materials and methods, as-synthesized octahedra characterization data, data from control and comparative experiments with benchtop measurements, EDX analysis, replicated data sets, further details from SEM, Raman, XPS, and XAS measurements, schematics of experimental setups and discussions regarding controls for identifying artifacts from the electron beam and cell geometry, beam effects in operando XAS measurements, and the quantification of ICP-MS results (PDF)
Movie tracking the evolution of the NiO octahedra during potential cycling as the Fe-containing electrolyte was flowed into the liquid cell (AVI)

## Supplementary Material

ja3c07158_si_001.pdf

ja3c07158_si_002.avi
