# Supplementary material for: Spatially and Chemically Resolved Visualization of Fe Incorporation into NiO Octahedra during the Oxygen Evolution Reaction
Source: J Am Chem Soc. 2023 Sep 19;145(39):21465–74. doi: 10.1021/jacs.3c07158 (PMC10557136; doi:10.1021/jacs.3c07158)
Supplement: Supplementary file 1 — ja3c07158_si_001.pdf [file ja3c07158_si_001.pdf]

Supporting Information

Spatially and Chemically Resolved Visualization of Fe Incorporation into NiO Octahedra during the Oxygen Evolution Reaction

Fengli Yang, Mauricio Lopez Luna, Felix T. Haase, Daniel Escalera-López, Aram Yoon, Martina Rüscher, Clara Rettenmaier, Hyo Sang Jeon, Eduardo Ortega, Janis Timoshenko, Arno Bergmann, See Wee Chee*, Beatriz Roldan Cuenya*

Affiliations

Department of Interface Science, Fritz-Haber-Institute of the Max-Planck Society, 14195, Berlin, Germany

*Corresponding authors: See Wee Chee (swchee@fhi-berlin.mpg.de) or Beatriz Roldan Cuenya (roldan@fhi-berlin.mpg.de).

This PDF file includes:

Materials and Methods

Supplementary Figures S1-S46

Supplementary Tables S1-S3

Supplementary Movie 1

Supporting Note 1-3

References 1-18

Materials and Methods

Synthesis of octahedral NiO

The synthesis of octahedral NiO catalysts follows the protocol described by Ma et al[1]. First, 2.5 g $NiCl_2 \cdot 6H_2O$ was loaded into a muffle furnace and was heated to 100°C for 3 hours. Subsequently, it was heated to 400°C and maintained at that temperature for another 2 hours. After cooling, the samples were washed several times with isopropyl alcohol and ultra-pure water. The final product was green in color.

Borate buffer electrolyte preparation and purification

In these experiments, a borate buffer solution (pH 9.8) is used as electrolyte instead of the conventional potassium hydroxide (KOH) solution to avoid possible silica contamination due to KOH etching of the microfabricated EC-TEM cells[2]. Performing OER using a borate buffer solutions as electrolyte is also better when employed in combination with photoabsorber materials due to its lower pH[2-3].

The 0.5 M borate buffer was prepared as follows. 31 g boric acid was dissolved in 1 L of ultra-pure water, and then 14 g NaOH were added into the above solution, resulting in a 0.5 M borate buffer solution with pH at ~9.8. To remove Fe impurities, the solution was purified according to the procedure described by Trotochaud et al.[4]. 2 g $Ni(NO_3)_2 \cdot 6H_2O$ were dissolved in 4 mL of 18.2 ultra-pure water, and then 20 mL of 1 M NaOH were added to obtain precipitated $Ni(OH)_2$. The mixture was washed with 200 mL of ultra-pure water and 20 mL of 1 M NaOH, and then centrifuged to obtain the supernatant. After that, the supernatant was added into 500 mL of prepared borate buffer for purification and kept for 3 h. Finally, the mixture was centrifuged to obtain the purified borate buffer supernatant.

Sample characterization

The crystallinity of the as-prepared NiO samples was characterized using X-ray diffraction (XRD), Bruker D8 Advance) with Cu Kα radiation. Elemental analysis of the catalysts to confirm the absence of Fe before reaction were performed using inductively coupled plasma mass spectrometry (ICPMS) combined with a microwave dilution system from Anton Paar (Multiwave GO). The XPS experiments were carried out with an ultrahigh vacuum (UHV) X-ray photoelectron spectroscopy/scanning tunneling microscopy (XPS/STM) system from SPECS (Germany) with a PHOIBOS 100 electron energy analyzer. A monochromatized Al Kα X-ray source (Focus 500) operated at 300 W was used for the measurements. The binding energy (BE) scale of the spectra was corrected taking the C-(C, H) component of the C 1s peak at 284.8 eV as a reference. The sample morphology was characterized with a Thermo Fisher Apreo scanning electron microscope (SEM). Transmission electron microscopy (TEM), high-resolution TEM (HRTEM), scanning TEM (STEM) and energy dispersive X-ray spectroscopy (EDX) elemental mapping and spectra of the octahedral NiO catalysts dropcasted on the TEM chip were obtained on a 300kV image-corrected (scanning) transmission electron microscope (Thermo Fisher Titan) with a TVIPS XF416R CMOS camera and a Bruker XFlash 6T|30 EDX spectrometer. Probe-corrected STEM imaging and electron energy-loss spectroscopy (EELS) was performed with a Jeol JEM-ARM200 that has a cold field emission gun and operated at 200 keV. The concurrent bright field (BF), annular dark field (ADF), and high-angle annular dark field images were recorded with a probe of 40 pA current and 15.6 mrad convergence angle. EELS data was acquired with a Gatan Quantum

ER imaging filter. The *ex situ* Raman was performed using a confocal Raman spectrometer (Renishaw, InVia Reflex) coupled with an optical microscope (Leica Microsystems, DM2500M). A near-infrared laser (Renishaw, RL633 λ=633 nm) was used as excitation source.

Electrochemical cell TEM (EC-TEM) experiments

The EC-TEM experiments were carried out in a Thermo Fisher Titan (S)TEM using a customized liquid flow cell setup from Hummingbird Scientific with a dedicated Ag/AgCl (3 M) reference and a carbon counter electrode. Figure S39 shows a schematic of the liquid cell TEM. For the experiments, a SP-200 potentiostat from Biologic was connected to the liquid cell TEM holder. The *in situ* experiments were carried out in STEM mode at 300 kV. Image sequences of the catalysts were acquired at a rate of 1 frame per second. Cyclic voltammetry (CV) and chronoamperometry (CA) measurements were performed in three-electrode configuration with the Ag/AgCl (3 M) reference and carbon counter electrodes. NiO octahedra were dropcasted on the electrochemistry chips purchased from Hummingbird Scientific with a carbon thin film as working electrode. Cyclic voltammetry and chronoamperometry measurements were performed in 0.5 M borate buffer (pH~9.8) with/without 1 mM $Fe(NO_3)_3$ solution added, respectively. Cyclic voltammetry was performed by applying a potential ranging from 0 to 1.3 V vs Ag/AgCl, scanning first in the positive direction at a sweep rate of 20 mV/s. The *ex situ* beaker experiments were performed with the octahedra dropcasted on carbon paper, glassy carbon supports or on EC-TEM chips. The measurements on carbon paper or glassy carbon are IR-corrected, but both *in situ/ex situ* measurements using the chips could not be corrected due to the small signal from the chips.

In situ imaging and the associated control experiments were performed according to the work already reported by our group[5]. *In situ* imaging was always performed with electrolyte in the cell, as determined from the image contrast, and we stayed under an electron flux of 7 e^{-} $Å^{-2}$ s^{-1} at all times to minimize electron beam-induced artifacts.

***Operando* Raman Spectroscopy**

The *operando* Raman experiments were performed using a confocal Raman spectrometer (Renishaw, InVia Reflex) coupled with an optical microscope (Leica Microsystems, DM2500M) and a home-built *operando* cell made of PTFE with a Ag/AgCl (3 M) reference and a Pt counter electrode[6]. Figure S40 shows a schematic of the *operando* Raman cell. A Biologic potentiostat (SP-240) was used to perform electrochemical measurements. A near-infrared laser (Renishaw, RL785 λ=785 nm, P_{max}=500mW) was used as excitation source. The backscattered light was Rayleigh-filtered and the Raman scattering was collected in the range of 75-1235 cm^{-1} with a grating of 1200 lines mm^{-1} and a CCD detector (Renishaw, Centrus). All Raman spectra were collected using a water inmersion objective (Leica Microsystems, 63x, Na 0.9) covered with a thin Teflon film (DuPont, 0.013 mm thickness) to avoid electrochemical attack from the reactive environment. The spectrometer was calibrated at 520.5 cm^{-1} using a Si(100) wafer.

X-ray absorption spectroscopy

Ni K-edge X-ray absorption spectroscopy (XAS) measurements at 8333.0 eV were performed at the KMC-3 beamline at BESSY II synchrotron in Berlin. Fe K-edge XAS data at 7112.0 eV were conducted at the P64 beamline at PETRA III at DESY in Hamburg. All measurements were conducted in fluorescence yield mode in a home-made electrochemical PEEK cell. The electrochemical setup consists of a leak-free Ag/AgCl reference electrode and Pt wire as counter

electrode. The catalysts were dropcasted on glassy carbon pieces (HTW) and the loading for all measurements was the same. Measurements were conducted in as-prepared state for the dry sample, at OCP after electrochemical conditioning (60 CVs, 0.8-2.0 V_{RHE}, 20 mV/s), during OER at 1.6 V_{RHE} and after OER at OCP. All displayed measurements were acquired at stationary conditions. Spectra were recorded for 30 minutes each and were averaged to improve the signal-to-noise ratio. Data were analyzed using the Athena software[7] and FEFFIT code[8].

ICP-MS experiments

H-cell experiments were performed using a three-electrode configuration, namely a hydrogen reference electrode (Mini HydroFlex, Gaskatel), a Pt mesh counter electrode (MatecK, 3600 mesh cm^{-2}) and a NiO thin film on a mirror-polished glassy carbon electrode (30mm*10mm, SIGRADUR). CE and RE/WE were placed in separate compartments, physically isolated by a membrane separator (Selemion AMV, AGC Inc.). The NiO film was obtained after dropcasting 3x2 µL of a 12.5 mg mL^{-1} aqueous NiO suspension (H_2O:IPA ratio of 3:1), yielding a catalyst spot loading of 0.93 mg_{cat} cm^{-2}. Purified borate buffer was used as is, whereas 1mM Fe purified borate buffer was pre-filtered (1 µm pore size) to remove non-dissolved Fe sediments. After filtration, effective Fe concentration decreased to ca. 0.5 mM according to ICP-MS quantification. Prior to any electrochemical experiment, all glassware and cell parts were sequentially cleaned to remove any organic/inorganic traces as follows: overnight soaking in a saturated $KMnO_4$ bath, washing with acidified H_2O_2 aqueous solution (removal of MnO_2 traces), soaking in freshly prepared aqua regia solution, and boiling in 3% HNO_3 (69% Supra, Roth).

Quantification of Fe uptake/loss in NiO octahedra was performed by replicating the EC-TEM experiments in a H-type cell configuration. During the H-cell experiments, a constant volume of 15 mL was employed per compartment: aliquots of 1 mL were withdrawn per each ICP-MS sample, followed by a 1mL injection of purified borate electrolyte to reinstate the initial total volume. Besides the samples being collected during cycling (0, 10, 20, 40, 60 and 120 CVs) and chronoamperometry (1mL every 10 mins) from the WE compartment, the electrolyte was also collected after electrochemical testing from the CE compartment to monitor Ni/Fe crossover. ICP-MS measurements were performed on a Thermo Scientific iCAQ RQ in KED mode (He flow rate: 0.65 L min^{-1}) using an ESI 2DX autosampler. Freshly prepared standard solutions containing known concentrations of Ni and Fe (from 0.5 to 50 µg L^{-1}, diluted from 10mg L^{-1} ICP-MS standards, Honeywell Flucka) were used to calibrate the ICP-MS, along with ^{59}Co (10 µg L^{-1}) as internal standard. Liquid sample dilution was performed before ICP-MS acquisition to satisfy a maximum tolerable total solid content of ca. 0.2 at. %.

Supplementary Figures and Tables

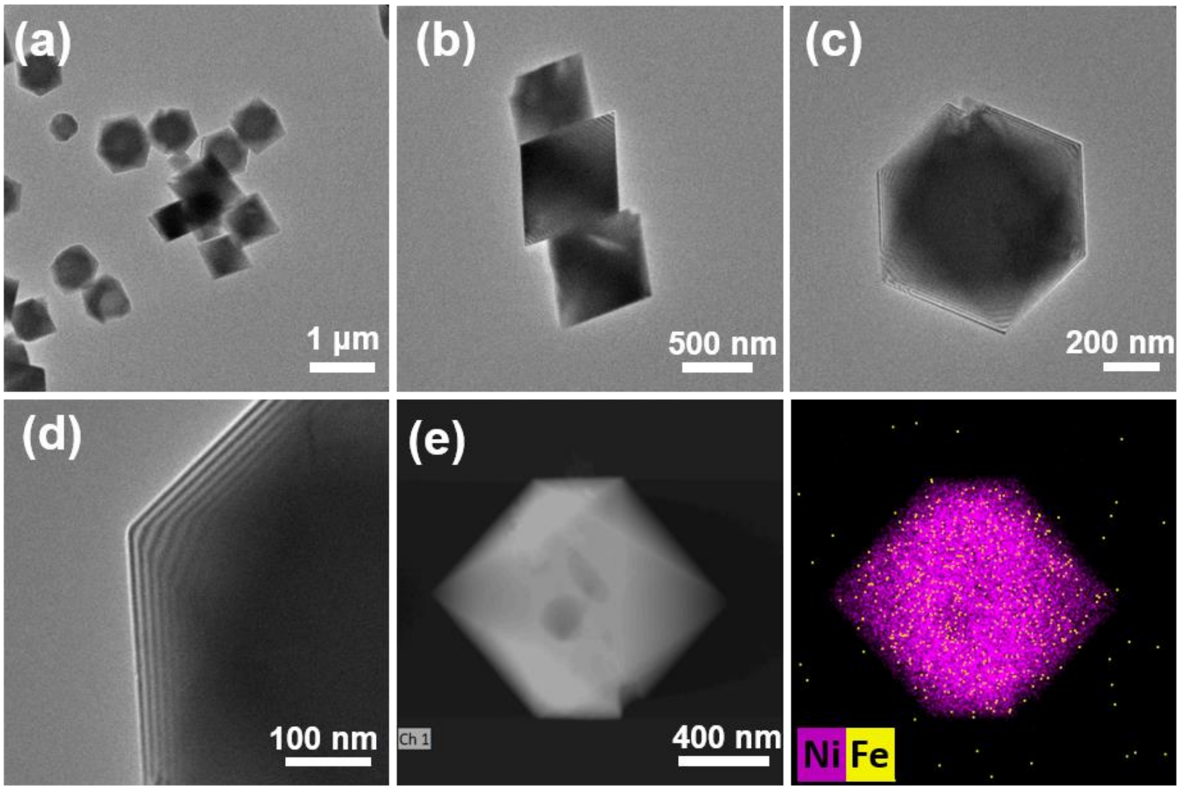

Figure S1. (a-d) *Ex situ* TEM images and (e) EDX map of an as-prepared NiO sample dropcasted on a carbon electrode chip. Because the Ni X-rays have sufficient energy to excite secondary emission of Fe X-rays, there is a very weak Fe signal in the EDX spectra acquired from the as-synthesized samples. This is an artifact that originates from the Fe present in the TEM column itself.

Table S1. Analytical results of as-prepared NiO octahedra by ICP-MS. The theoretical Ni concentration is based on a digested mass of 0.79 mg.

Sample	Fe Concentration (ug/L)	Ni Concentration (ug/L)	Fe/Ni Molar Ratio (%)
**NiO Octahedra Powder**	28.46	82558.73	0.03

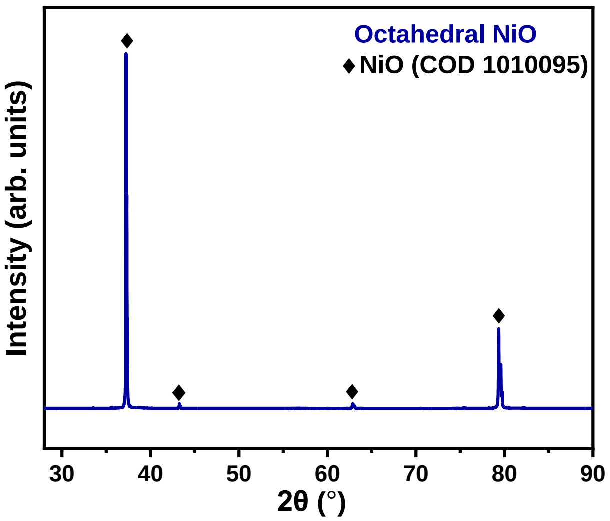

Figure S2. XRD pattern of as-prepared NiO sample.

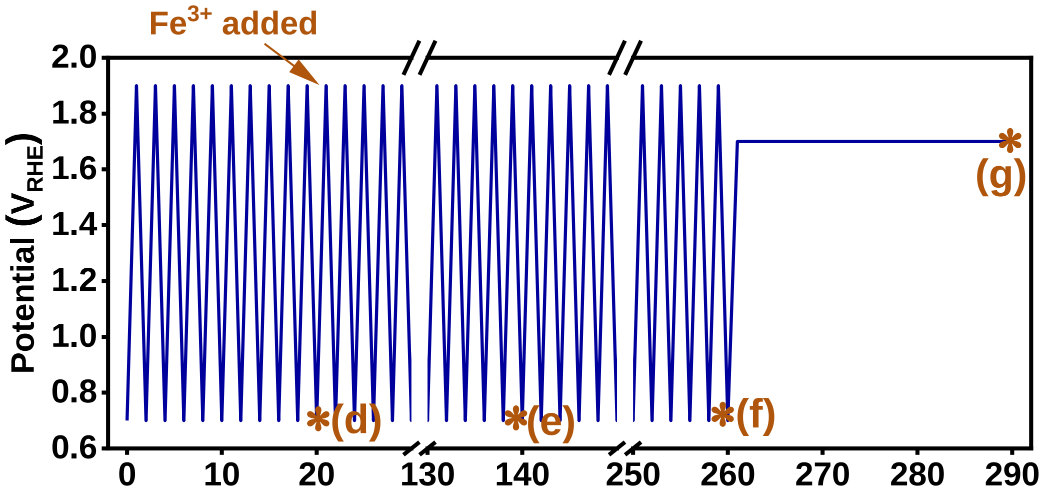

Figure S3. Graphical illustration showing the applied potential waveform during a cyclic voltammetry measurement. The depicted time points (d-g) in the figure show the time to collect *in situ* EDX in EC-TEM cell during the experiment, as shown in Figure 1 in the manuscript. (d) Before applying the potential, (e) after 60 cycles of CVs, (f) after 120 cycles of CV from 0.7 to 1.9 V_{RHE} and (g) after 120 cycles of CV scanning and the chronoamperometric measurements for 30 minutes at 1.7 V_{RHE}.

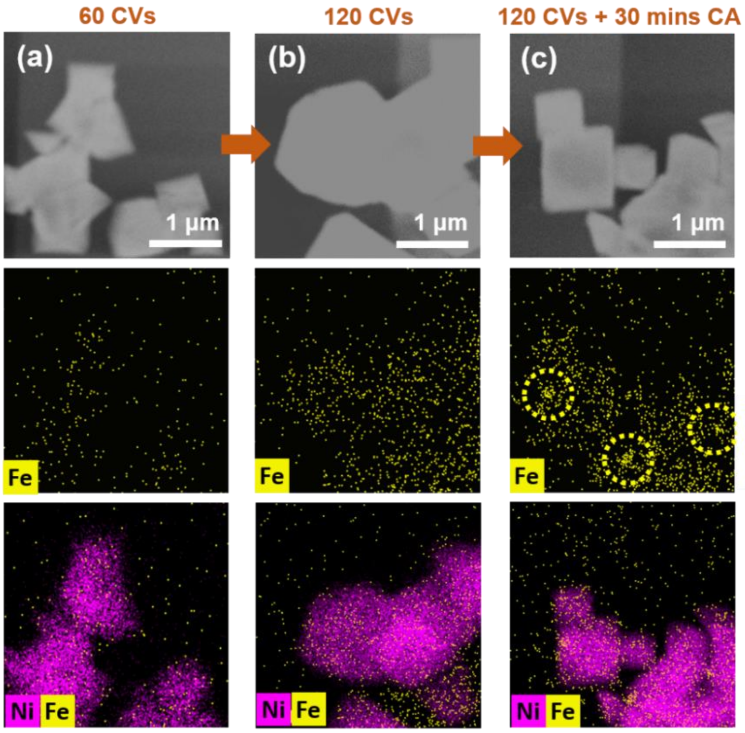

Figure S4. (a)-(c) Additional *in situ* STEM images and combined Ni/Fe chemical EDX maps of NiO octahedral acquired in a 0.5 M borate buffer +1 mM $Fe(NO_3)_3$ solution. (a) after 60 cycles of CV from 0.7 to 1.9 V_{RHE} and (b) after 120 cycles of CV from 0.7 to 1.9 V_{RHE}. (c) STEM image and Fe map collected after an additional 30 minutes at 1.7 V_{RHE} after 120 CV cycles. Aggregated Fe is highlighted in (c) with dashed circles. The EDX maps in (a-c) are collected under applied potential.

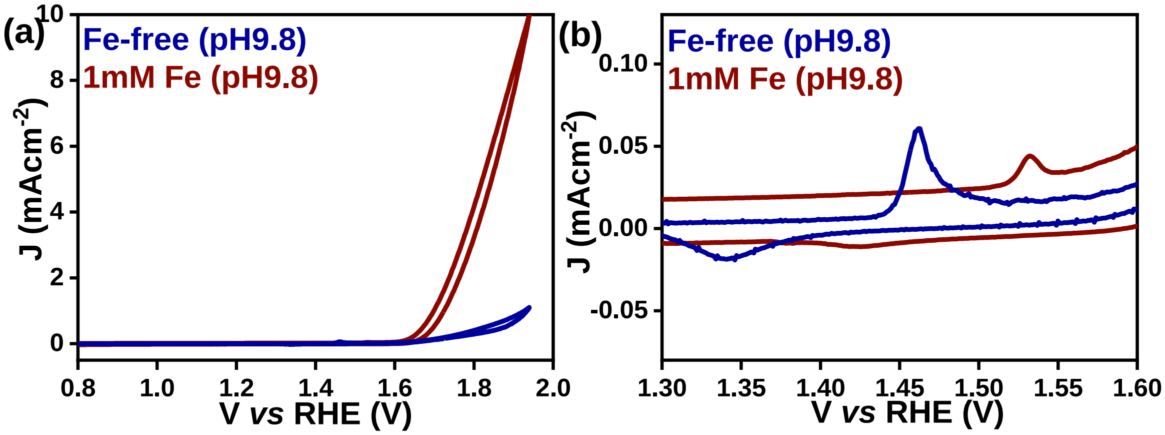

Figure S5. (a) A comparison of cyclic voltammograms acquired in 0.5 M borate buffer without Fe after 10 cycles and acquired after 40 cycles with 1 mM $Fe(NO_3)_3$ added into the electrolyte flow using EC-TEM. (b) An enlarged display of (a) for the potential range between 1.3 to 1.6 V_{RHE}. The NiO octahedra were dropcasted on a carbon electrode chip.

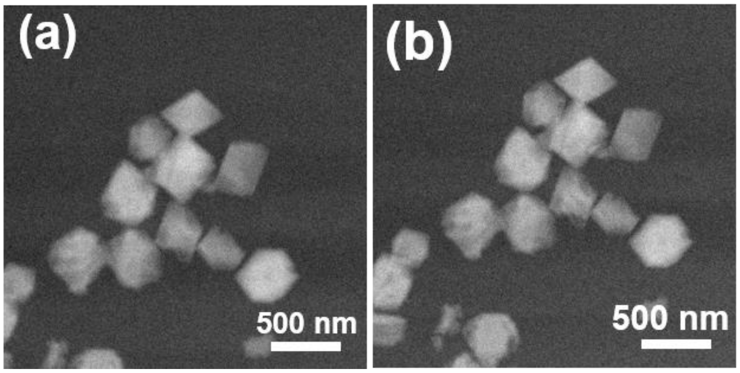

Figure S6. *In situ* STEM images of the NiO catalysts in (a) 0.5 M borate buffer solution and (b) 0.5 M borate buffer +1 mM $Fe(NO_3)_3$ solution during OER from 0.7 to 1.9 V_{RHE}.

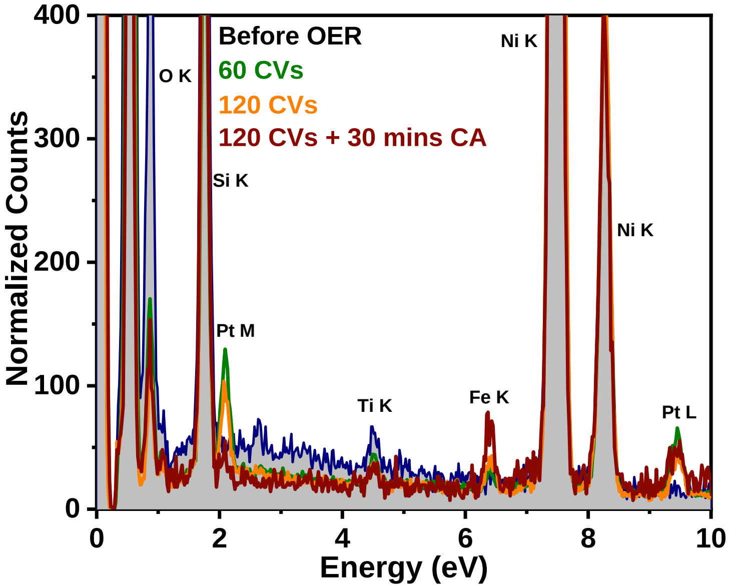

Figure S7. *In situ* EDX spectra of NiO catalysts before OER, after 60 cycles of CV scanning, after 120 cycles of CV scanning and after 120 cycles of CV scanning and the chronoamperometric measurements for 30 minutes at 1.7 V_{RHE} in 0.5 M borate buffer +1 mM $Fe(NO_3)_3$ solution using EC-TEM cell. The CV scanning was performed in the potential window from 0.7 to 1.9 V_{RHE} at a scan rate of 20 mV s^{-1}. The spectra are normalized to the Ni K peak of the NiO. The Pt signal comes from the EC-TEM chips. In these cells, the Pt contacts are first lithographically patterned on the silicon nitride surface after which the contacts are overlaid with the carbon film[9], so the Pt contacts don't affect the reaction, just contribute to the conductivity. The Ti signal originates from the electrochemical cell TEM holder.

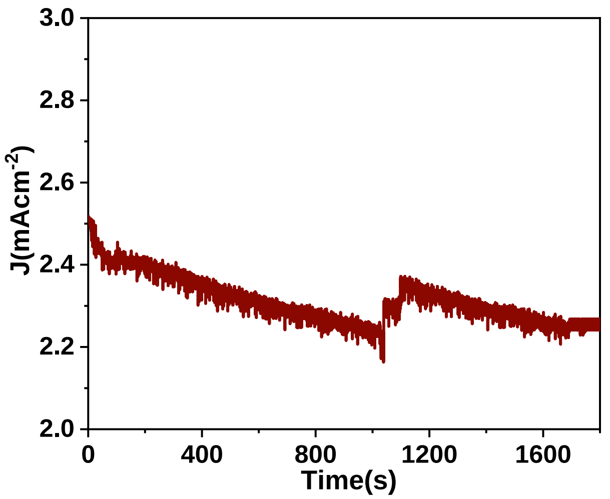

Figure S8. Chronoamperometric response obtained for NiO at 1.7 V_{RHE} in 0.5 M borate buffer + 1 mM $Fe(NO_3)_3$ solution using EC-TEM cell after 120 cycles of CV. The NiO octahedra were dropcasted on a carbon electrode chip.

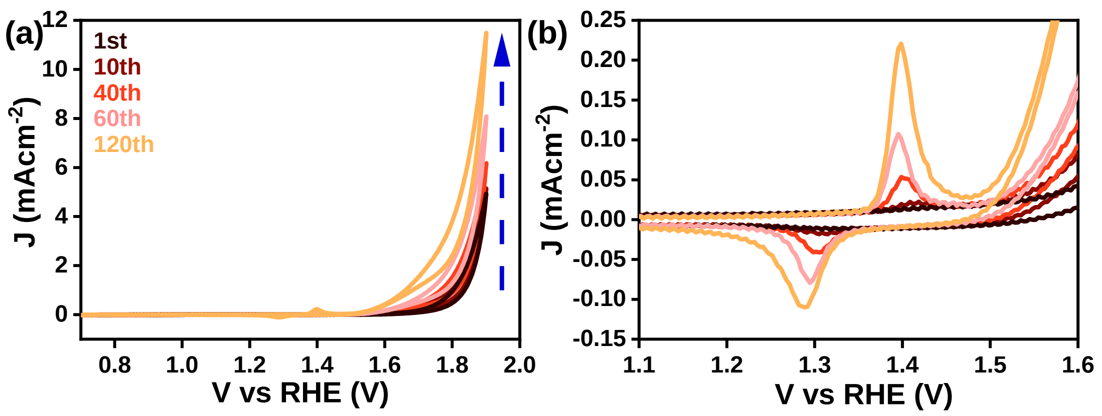

Figure S9. (a) Cyclic voltammograms acquired with the NiO octahedra as electrocatalysts in 0.5 M borate buffer without Fe using a standard benchtop electrochemistry setup. The NiO octahedra were dropcasted on a carbon electrode chip. The slow increase in the anodic current is attributed to the activation of the electrocatalysts. (b) An enlarged display of (a) for the potential range between 1.1 to 1.6 $V_{RHE.}$

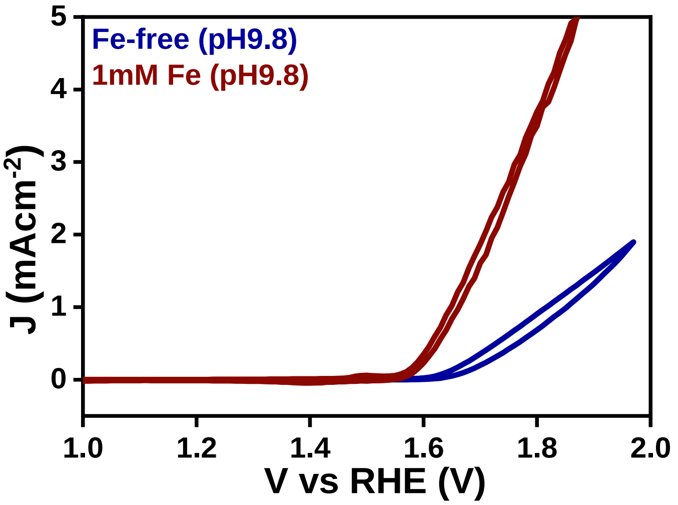

Figure S10. Cyclic voltammograms acquired with the NiO octahedra as electrocatalysts in 0.5 M borate buffer without/with Fe using our standard benchtop electrochemistry setup. All potentials are IR-corrected. The NiO octahedra were dropcasted on carbon paper.

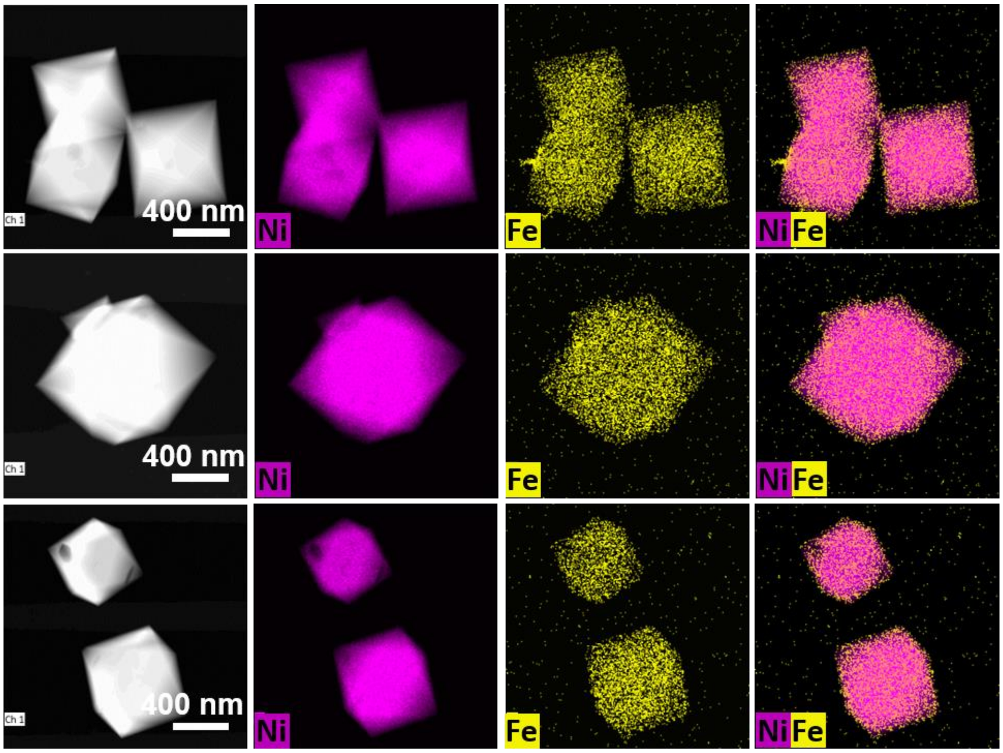

Figure S11. *Ex situ* STEM-EDX images of NiO octahedra after 40 cycles of CV scanning in 0.5 M borate buffer +1 mM $Fe(NO_3)_3$ solution. All CVs were acquired within the same potential window from 0.7 to 1.9 V_{RHE} at a scan rate of 20 mV s^{-1} in EC-TEM cell.

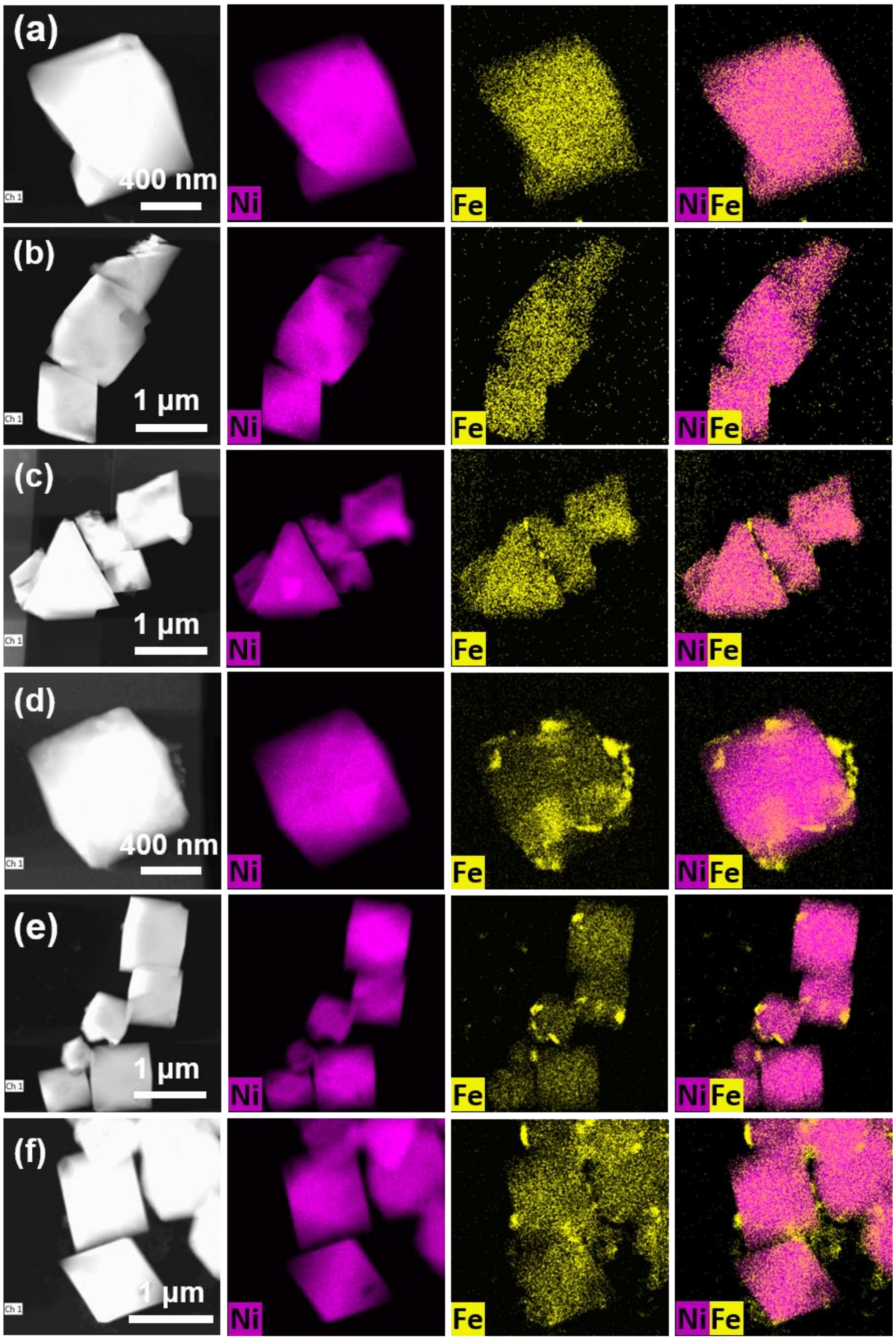

Figure S12. *Ex situ* EDX maps showing elemental distribution of Ni and Fe in NiO catalysts after *in situ* EDX experiments under different reaction conditions. (a) and (b) after 60 cycles of CV scanning, (c) after 120 cycles of CV scanning and (d-f) after 120 cycles of CV scanning and the chronoamperometric measurements for 30 minutes at 1.7 V_{RHE} in 0.5 M borate buffer +1 mM $Fe(NO_3)_3$ solution. All the CVs are at the same potential window from 0.7 to 1.9 V_{RHE} at 20 $mV{\cdot}s^{-1}$ scan rate in EC-TEM cell.

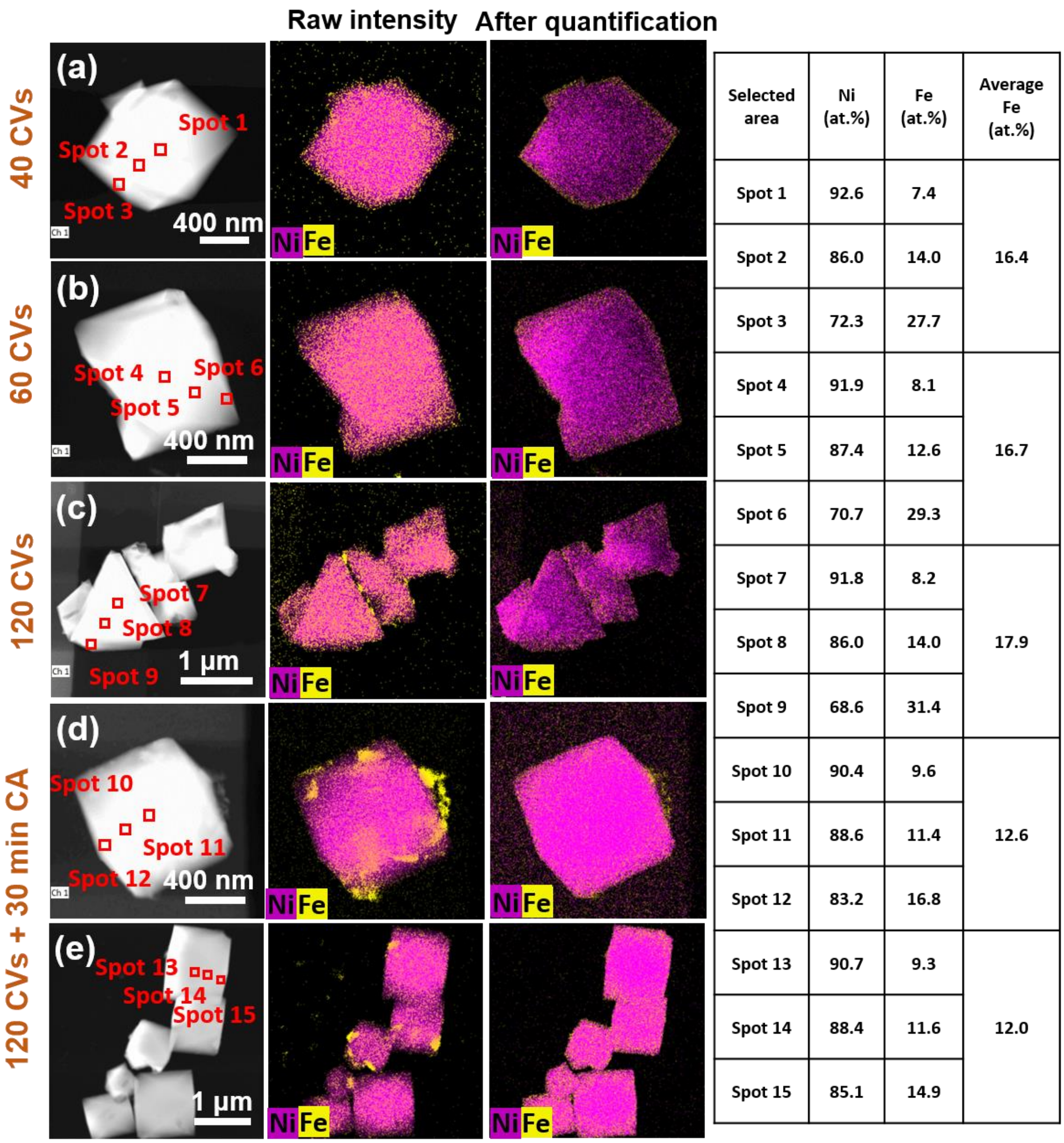

Selected area	Ni (at.%)	Fe (at.%)	Average Fe (at.%)
Spot 1	92.6	7.4	
Spot 2	86.0	14.0	16.4
Spot 3	72.3	27.7	
Spot 4	91.9	8.1	
Spot 5	87.4	12.6	16.7
Spot 6	70.7	29.3	
Spot 7	91.8	8.2	
Spot 8	86.0	14.0	17.9
Spot 9	68.6	31.4	
Spot 10	90.4	9.6	
Spot 11	88.6	11.4	12.6
Spot 12	83.2	16.8	
Spot 13	90.7	9.3	
Spot 14	88.4	11.6	12.0
Spot 15	85.1	14.9	

Figure S13. *Ex situ* STEM images, EDX intensity maps and EDX Ni/Fe atomic percentage maps of NiO catalysts after *in situ* EDX experiments in 0.5 M borate buffer + 1 mM $Fe(NO_3)_3$ solution under different reaction conditions and point scan results: (a) after 40 cycles of CV scanning, (b) after 60 cycles of CV scanning, (c) after 120 cycles of CV scanning and (d,e) after 120 cycles of CV scanning and chronoamperometric measurements for 30 minutes at 1.7 V_{RHE}. The STEM images highlight the areas where the atomic percentage of Ni and Fe is calculated.

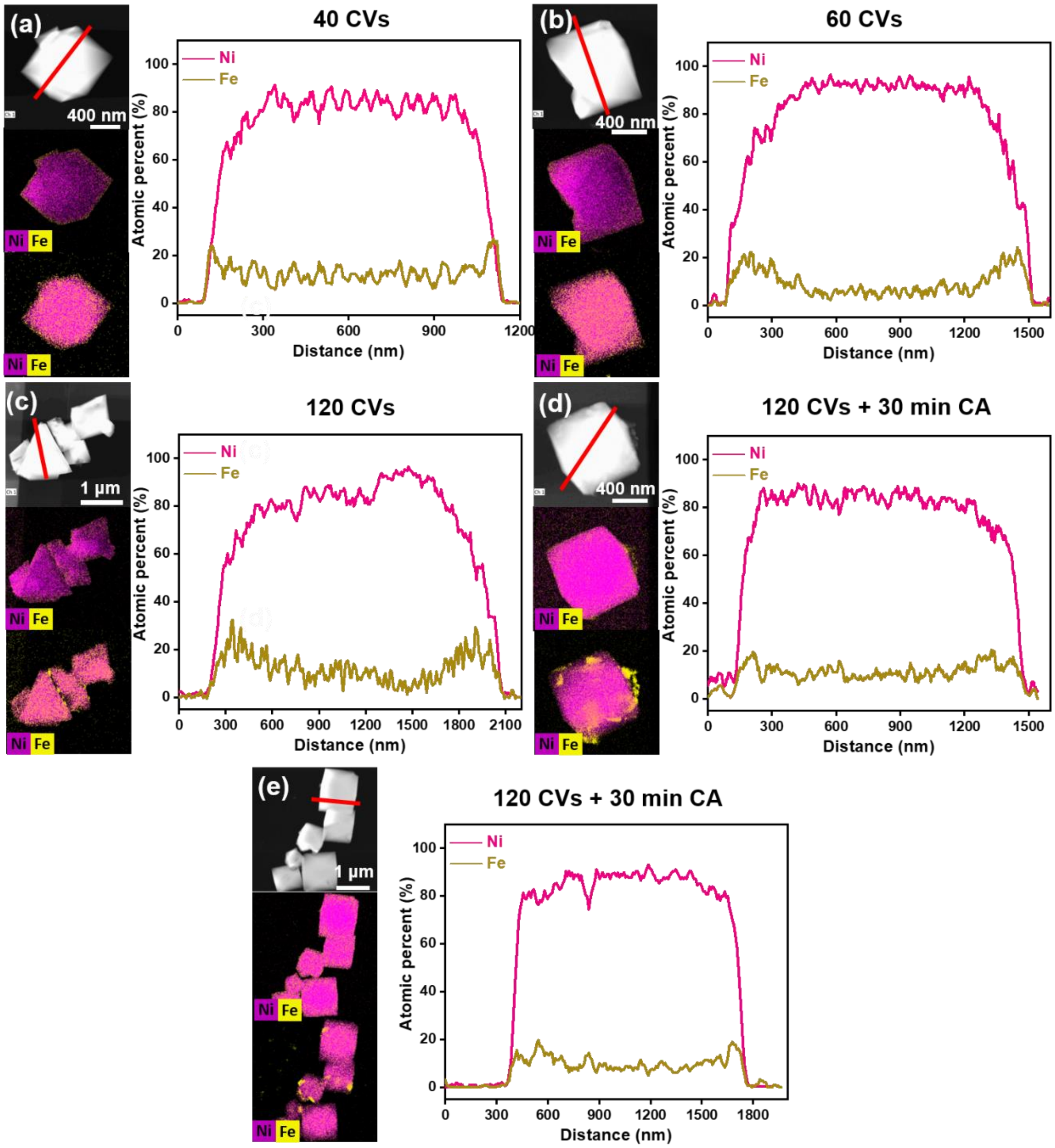

Figure S14. *Ex situ* EDX Ni/Fe atomic percentage line scans of NiO catalysts after *in situ* EDX experiments in 0.5 M borate buffer + 1 mM $Fe(NO_3)_3$ solution under different reaction conditions and line scan results: (a) after 40 cycles of CV scanning, (b) after 60 cycles of CV scanning, (c) after 120 cycles of CV scanning and (d,e) after 120 cycles of CV scanning and chronoamperometric measurements for 30 minutes at 1.7 V_{RHE}. The red line in the reference STEM images indicates the path selected to identify the elemental composition of the surface layer and core.

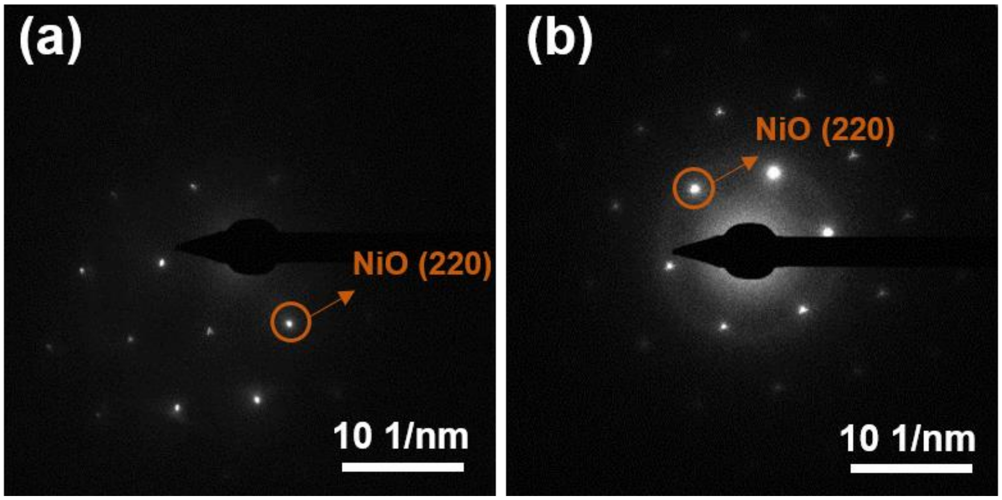

Figure S15. SAED images of as-prepared NiO sample (a) before and (b) after 120 cycles of CV scanning and the chronoamperometric measurements for 30 minutes at 1.7 V_{RHE} in 0.5 M borate buffer + 1 mM $Fe(NO_3)_3$ solution. The sample was dropcasted on a carbon electrode chip.

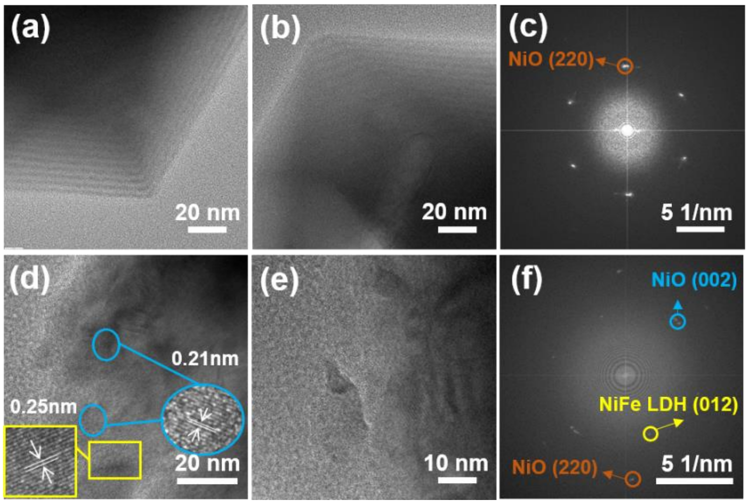

Figure S16. HRTEM and FFT images of the NiO samples. (a-c) before OER, (d-f) after 120 cycles of CV scanning and the chronoamperometric measurements for 30 minutes at 1.7 V_{RHE}. The sample was dropcasted on a carbon electrode chip.

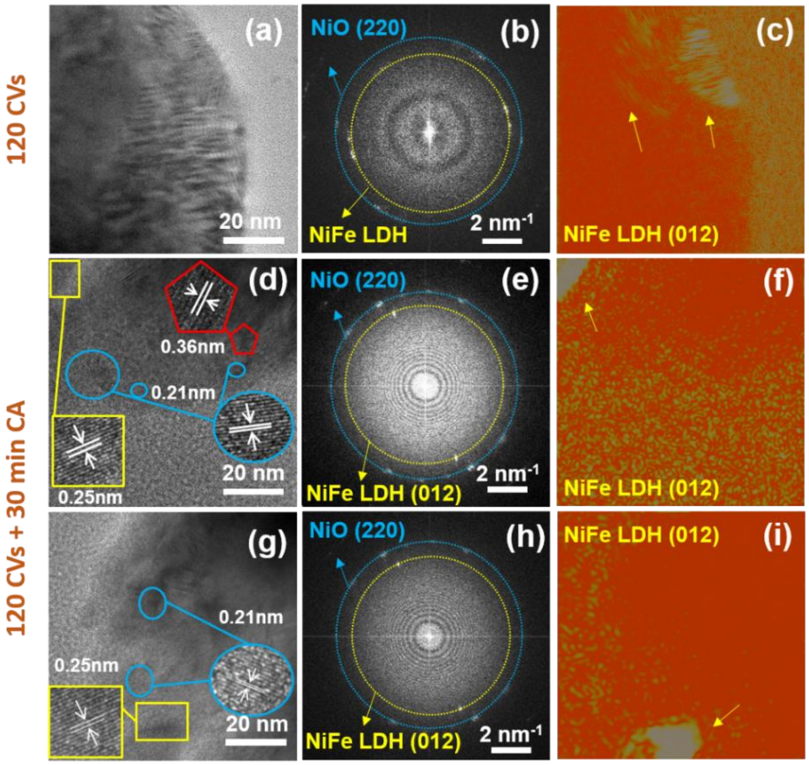

Figure S17. (a,d,g) HRTEM images, (b,e,h) FFT, and (c,f,i) inversed FFT obtained by Digital Micrograph by masking the 012 spots of NiO: (a-c) after 120 cycles of CV scanning and (d-i) after 120 cycles of CV scanning and chronoamperometric measurements for 30 minutes at 1.7 V_{RHE}. The lattice spacing was measured in both HRTEM and the corresponding frequency domain images.

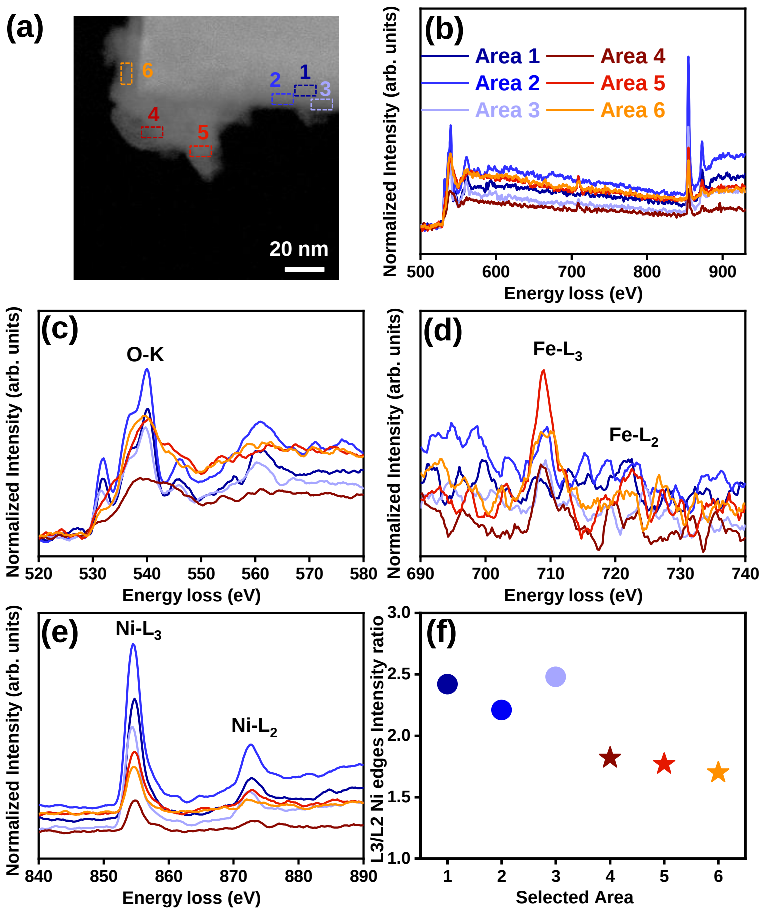

Figure S18. (a) STEM-ADF image of a Ni-Fe LDH flake on a NiO octahedron. The colored rectangles highlight the regions where (b) the EELS spectra were recorded (background subtracted data shown). The sample was exposed to 60 cycles of CV scanning in 0.5 M borate buffer + 1mM $Fe(NO_3)_3$ solution using our standard benchtop electrochemistry setup. The sample was dropcasted on a glassy carbon support and then transferred onto a standard TEM grid after reaction**.** (c) K-edge of O obtained from the flake (4-6) and the NiO surface (1-3). In NiO, three main peaks are found at about 531.5eV, 539.8 eV and 560.2 eV, respectively. These features are absent from the O edge extracted from the flakes. Background-subtracted (d) Fe and (e) Ni $L_{3,2}$ edges of flake both show white-line features, whereas the NiO only show Ni white-line features. The L_3 and L_2 correspond to excitations from the spin-orbit slit levels $2p_{3/2}$ and $2p_{1/2}$ and can be used to infer the oxidation state differences based on their total integral intensity ratio (L_3/L_2). The peaks of the $L_{3,2}$ edges of Fe and Ni are located at around 708.8 and 722.4 and 854.6 and 872.7 eV, respectively. For (d) Fe, we cannot determine the oxidation due to the relatively noisy spectra but the absence of a doublet at L_3 edge of Fe indicate there is no co-existing Fe^{2+} and Fe^{3+}. On the other hand, the L_3/L_2 ratios of Ni ((e)-(f)) in flakes is lower than that in NiO, indicating that Ni exists in a higher oxidation state.

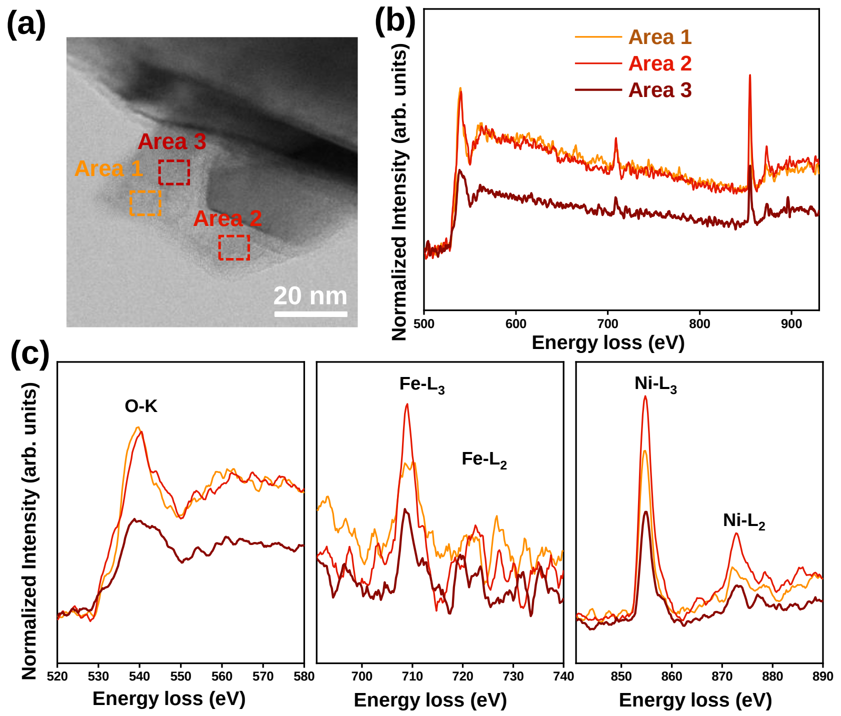

Figure S19. (a) STEM-BF image of another NiFe-LDH flake. The red rectangles highlight the region where (b) EELS spectra were recorded (background subtracted data shown). (c) The O, Fe and Ni edges obtained from the flake. The sample was exposed to 60 cycles of CV scanning in 0.5 M borate buffer + 1mM $Fe(NO_3)_3$ solution using our standard benchtop electrochemistry setup. The sample was dropcasted on a glassy carbon support and then transferred onto a standard TEM grid after reaction.

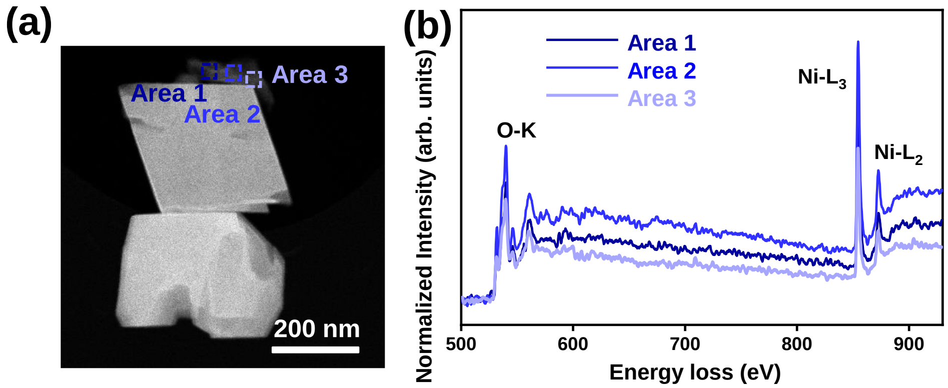

Figure S20. (a) STEM-ADF image of NiO structures. The red rectangles highlight the region where (b) EELS spectra were recorded (background subtracted data shown). The reference sample without Fe was exposed to 120 cycles of CV scanning in 0.5 M borate buffer solution using our standard benchtop electrochemistry setup. The sample was dropcasted on a glassy carbon support and then transferred onto a standard TEM grid after reaction.

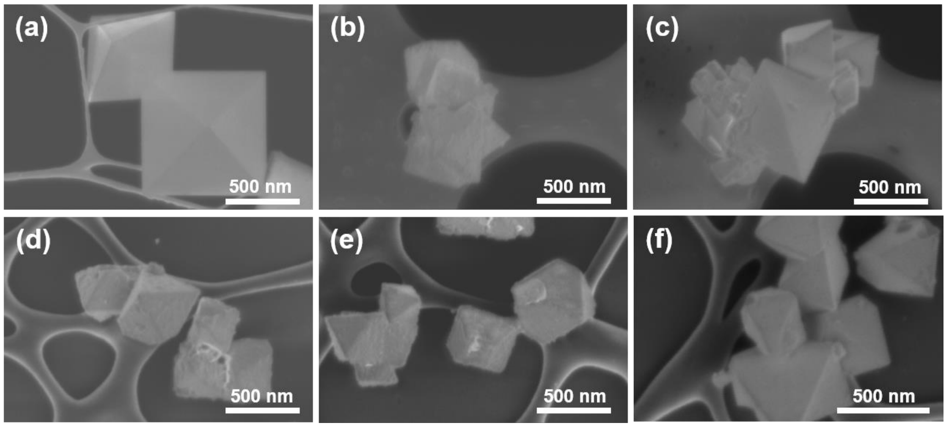

Figure S21. *Ex situ* SEM images of the NiO samples. (a) before OER, (b) after 120 cycles of CV scanning in 0.5 M borate buffer solution, (c) after 60 cycles of CV scanning, (d,e) after 120 cycles of CV scanning and (f) after 120 cycles of CV scanning and chronoamperometric measurements for 1 hour at 1.7 V_{RHE} in 0.5 M borate buffer +1 mM $Fe(NO_3)_3$ solution using our standard benchtop electrochemistry setup. The sample was dropcasted on a glassy carbon support and then transferred onto a standard TEM grid after reaction.

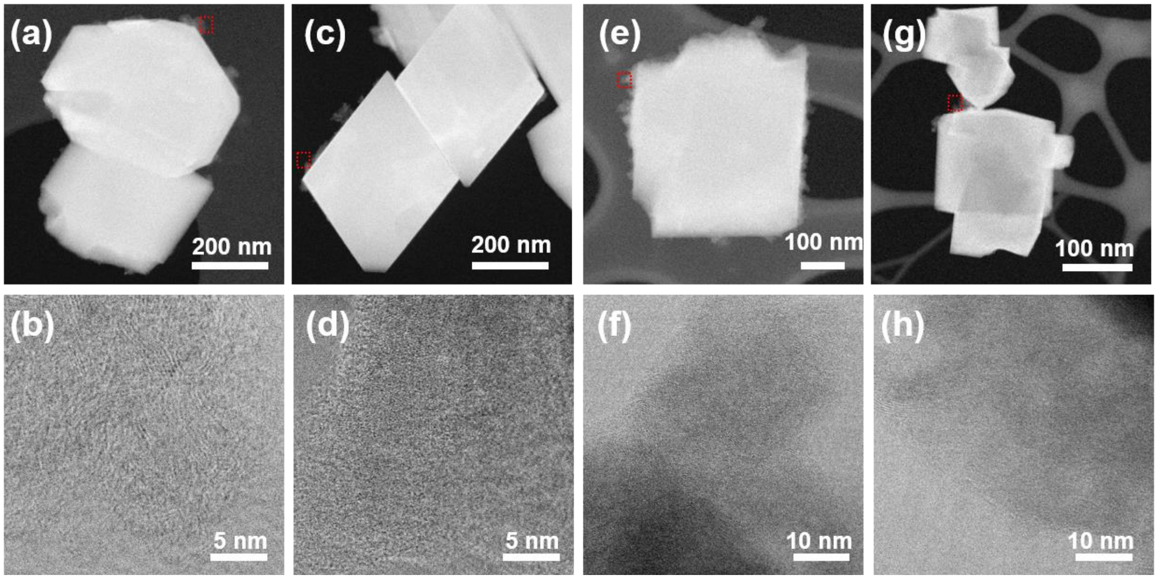

Figure S22. ADF images of NiO samples. (a,b) after 120 cycles of CV scanning in 0.5 M borate buffer solution, (c,d) after 60 cycles of CV scanning, (e,f) after 120 cycles of CV scanning and (g,h) after 120 cycles of CV scanning and chronoamperometric measurements for 1 hour at 1.7 V_{RHE} in 0.5 M borate buffer +1 mM $Fe(NO_3)_3$ solution using our standard benchtop electrochemistry setup. (b, d, e, f) are magnified images of the surface flakes showing their poorly ordered nature. The sample was dropcasted on a glassy carbon support and then transferred onto a standard TEM grid after reaction.

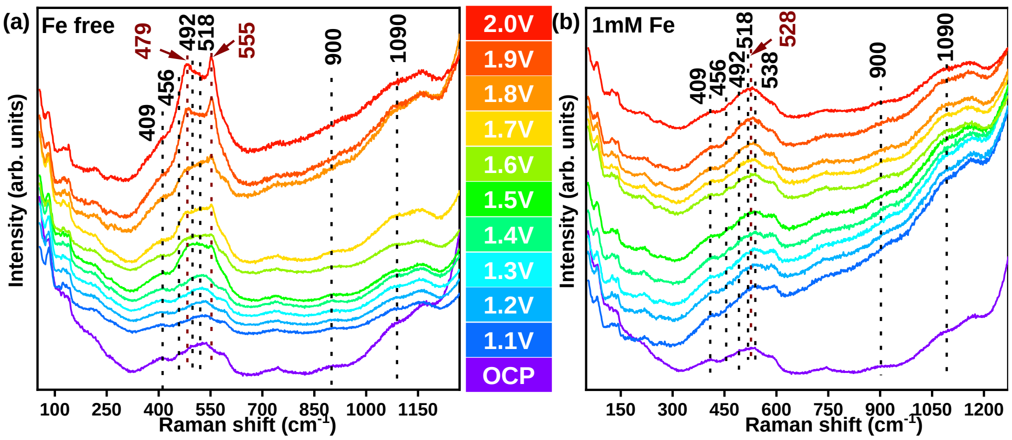

Figure S23. *Operando* Raman spectra collected as the potential was increased from 1.0 V to 2.0 V_{RHE} in (a) 0.5 M borate buffer and (b) 0.5 M borate buffer + 1 mM $Fe(NO_3)_3$ solution. The measurements were performed at a fixed potential.

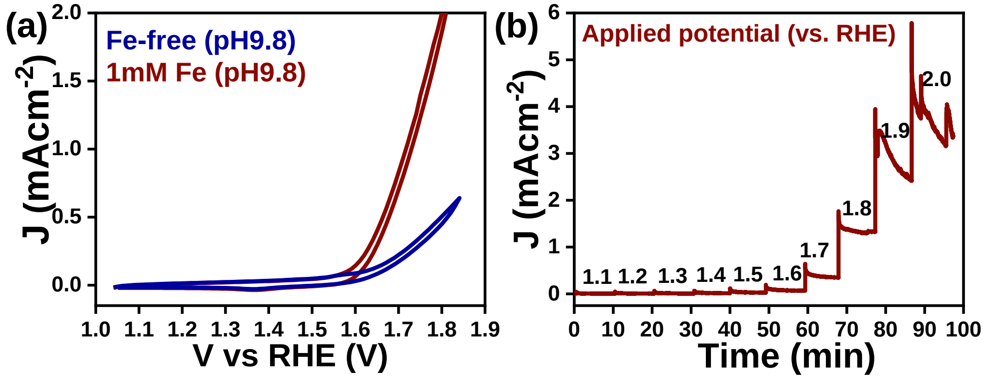

Figure S24. (a) The result of cyclic voltammetry measurements for as-prepared NiO using *operando* electrochemical Raman cell in different electrolytes. (b) The result of chronoamperometric measurements for as-prepared NiO using *operando* electrochemical Raman cell in 0.5 M borate buffer + 1 mM $Fe(NO_3)_3$ solution. All potentials are IR-corrected.

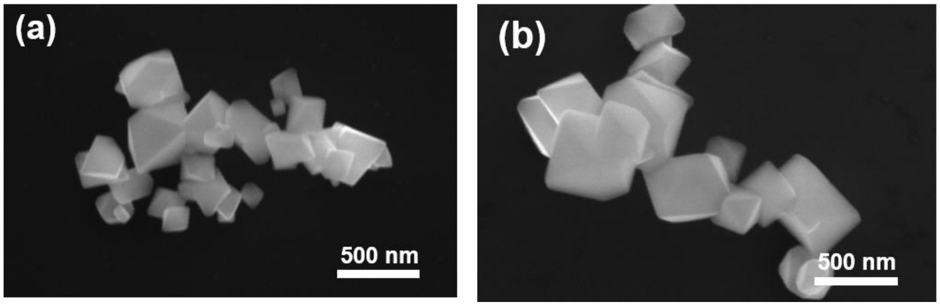

Figure S25. SEM images of NiO sample dropcasted on a glassy carbon (a) before and (b) after *operando* Raman experiments in 0.5 M borate buffer + 1 mM $Fe(NO_3)_3$ solution.

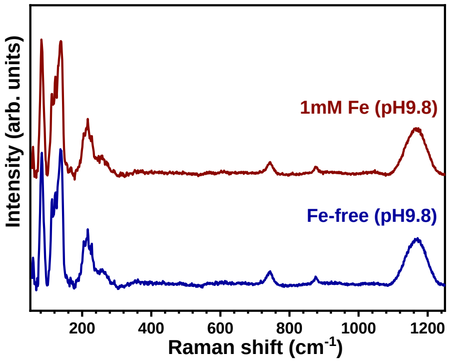

Figure S26. *Operando* Raman spectra collected in 0.5 M borate buffer and 0.5 M borate buffer + 1mM Fe(NO3)3 solution without NiO catalyst.

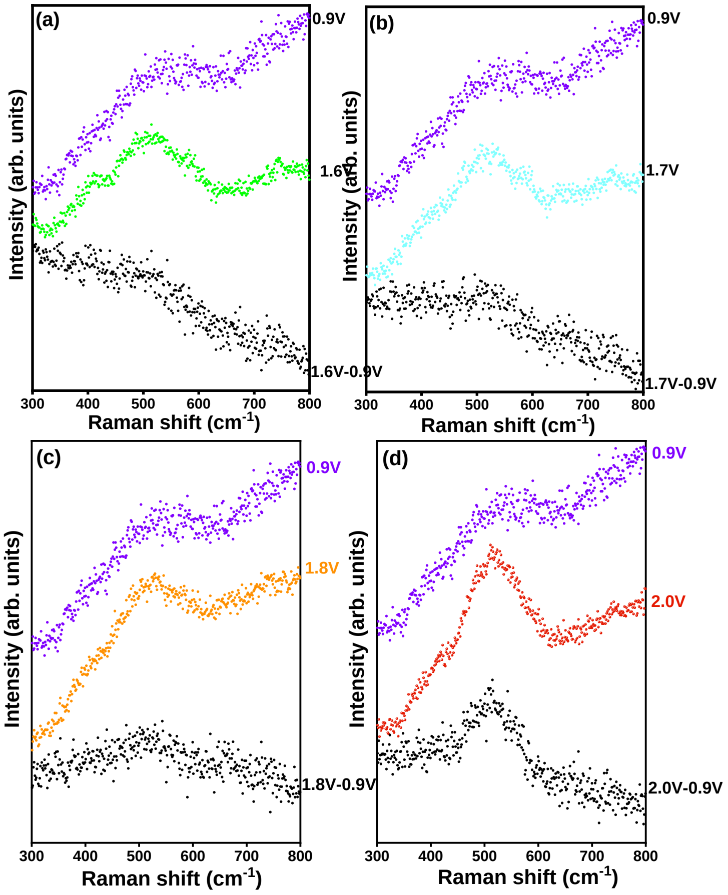

Figure S27. *Operando* Raman spectra normalized based on the electrolyte band at 83 cm^{-1}. Baseline subtraction was performed by fitting function to a polynomial baseline with order 4 and 1.5 noise tolerance using WIRE 5.2 from Renishaw. The black difference spectra are the result of the subtraction of the spectra at 0.9 V $_{RHE}$ (blue) from the one at 1.6 V_{RHE} (green), the one at 1.7 V_{RHE} (blue), the one at 1.8 V_{RHE} (orange) and the one at 2.0 V_{RHE} (red) in 0.5 M borate buffer + 1 mM Fe(NO3)3 solution.

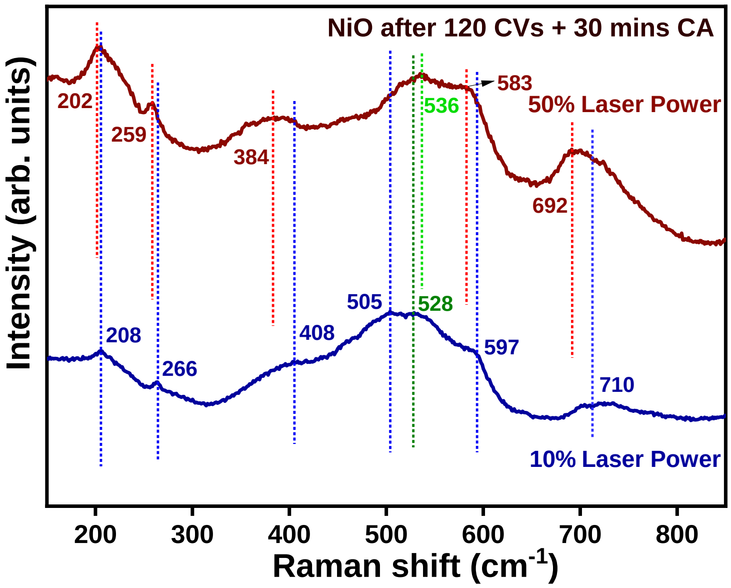

Figure S28. *Ex situ* Raman spectroscopy of the NiO octahedra dropcasted on glassy carbon after 120 cycles of CV scanning and the chronoamperometric measurements for 30 minutes at 1.7 V_{RHE} in 0.5 M borate buffer + 1 mM $Fe(NO_3)_3$ solution obtained under different laser power. The CV scanning was performed in the potential window from 0.7 to 1.9 V_{RHE} at a scan rate of 20 mV s^{-1}. Under lower laser power, the broad peak of the spectrum at 528 cm^{-1} matches the previous *operando* Raman data and prior data available in the literature from NiFe LDH films[10]. The peaks at 208, 266, 408, 505 and 597 cm^{-1} can be assigned to α-Fe_2O_3[11-13]. There is a downshift for the bands in comparison to the bulk crystalline α-Fe_2O_3 that might be assigned to the poor crystallinity of our hematite, which is present as small clusters according to the local TEM/EDX measurements. The broad peak at 710 cm^{-1} can be explained by a phase change to maghemite (γ-Fe_2O_3) induced by the intense Raman laser[11], which was further corroborated in the spectrum acquired under higher laser power.

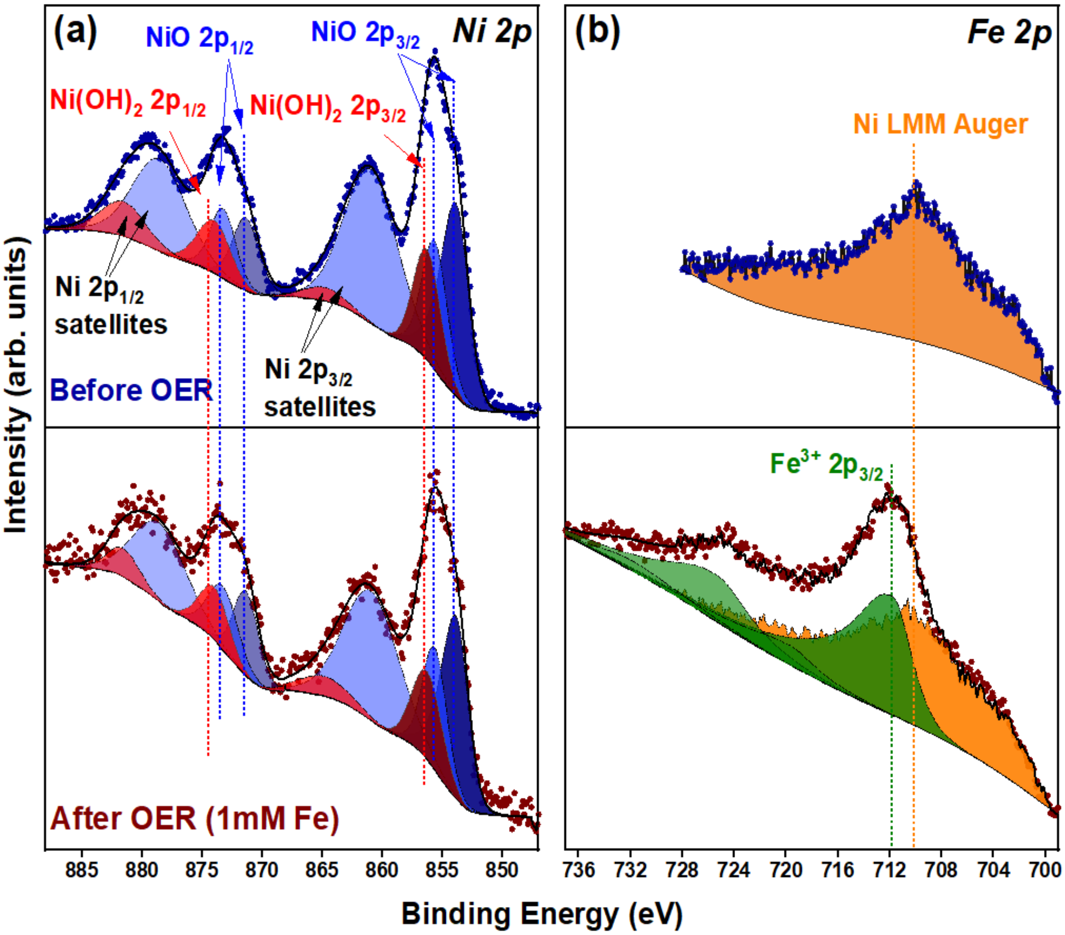

Figure S29. *Ex situ* XPS spectra of the (a) the Ni 2p region and (b) Fe 2p region of the NiO catalyst before catalysis and after chronoamperometric measurements for 60 minutes at 1.7 V_{RHE} in 0.5 M borate buffer + 1 mM $Fe(NO_3)_3$ solution. The octahedral NiO catalysts were dropcasted on carbon paper, and a benchtop setup was used as the reaction cell. The reference electrode is Ag/AgCl, and the counter electrode is carbon. The components fitted in the Ni $2p_{3/2}$ region are NiO (853.8 eV, fit component shown in dark blue and 855.8 eV, fit component shown in light blue), and $Ni(OH)_2$ (856.3eV, fit component shown in dark red)[14-15]. Fitting of the Ni 2p region indicates negligible differences in the Ni composition before and after reaction, with both showing NiO (79.3%) and $Ni(OH)_2$ (20.7%). Due to the overlap of the Fe-2p with the Ni LMM Auger signal, the fingerprint from this Ni LMM was fitted into the Fe-2p region for quantification of the Fe-2p contribution. The components fitted into the Fe-2p region are related to Fe^{3+} (711.8 eV)[16]. Fe^{3+} could indicate hematite or maghemite, or a NiFe LDH. The experimental Ni: Fe atomic ratio found for the surface composition of the after OER samples is 2.7:1, which is close to the 3:1 ratio commonly reported for NiFe LDH[10].

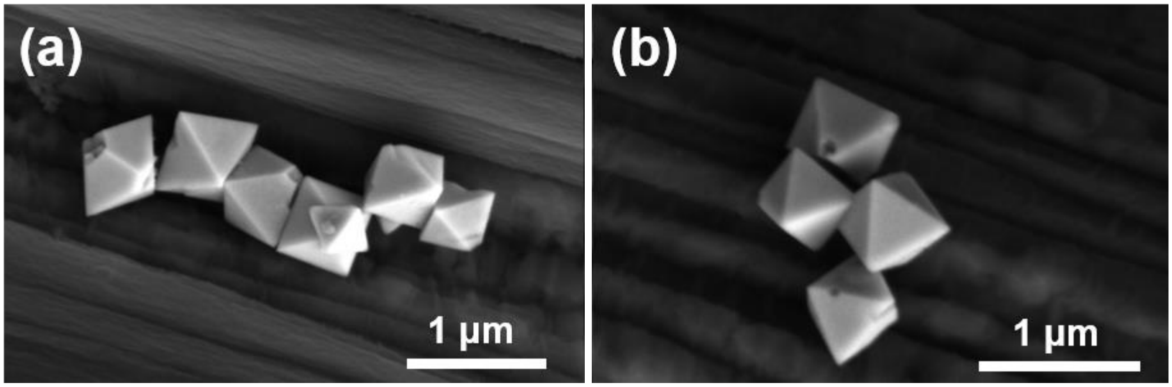

Figure S30. The SEM images of NiO sample dropcasted on carbon paper (a) before and (b) after the chronoamperometric measurements for 60 minutes at 1.7 V_{RHE} in 0.5 M borate buffer + 1 mM $Fe(NO_3)_3$ solution used for XPS measurements.

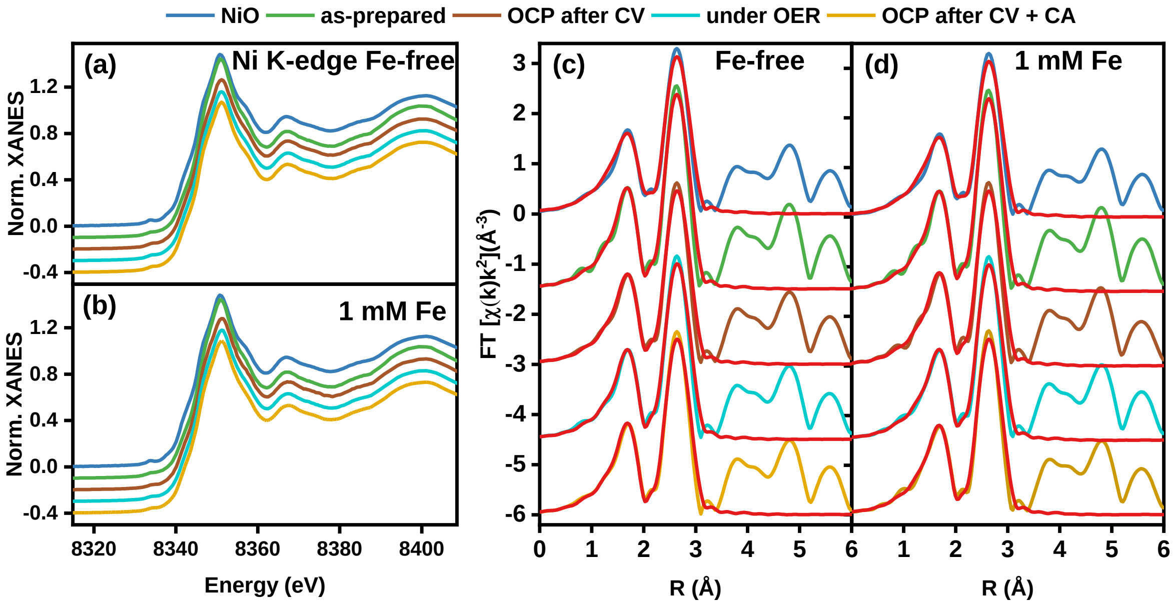

Figure S31. Vertically displaced normalized Ni K-edge XANES data for catalyst in (a) 0.5 M borate buffer solution and (b) 0.5 M borate buffer + 1 mM $Fe(NO_3)_3$ solution. Fourier-transformed extended X-ray absorption fine structure (FT-)EXAFS) collected at the Ni K-edge for catalyst in (c) 0.5 M borate buffer solution and (d) 0.5 M borate buffer + 1 mM $Fe(NO_3)_3$ solution. Fits of the EXAFS spectra are shown in red and the spectra are stacked vertically. Measurements were performed for as-prepared samples in a dry state, activated samples after conditioning, during OER and after OER. Corresponding data recorded for NiO reference material are also shown for comparison.

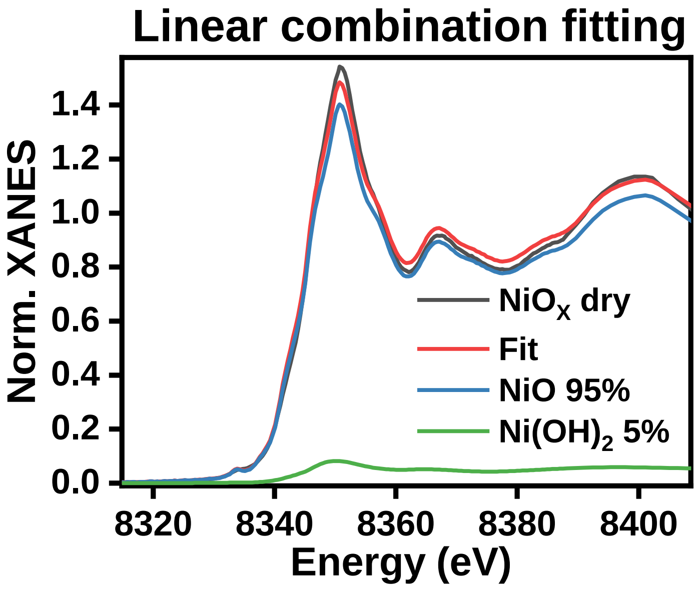

Figure S32. Linear combination fitting (LCF) of Ni K-edge XANES spectra for NiO_X octahedra, using XANES spectra for NiO and $Ni(OH)_2$ as reference spectra.

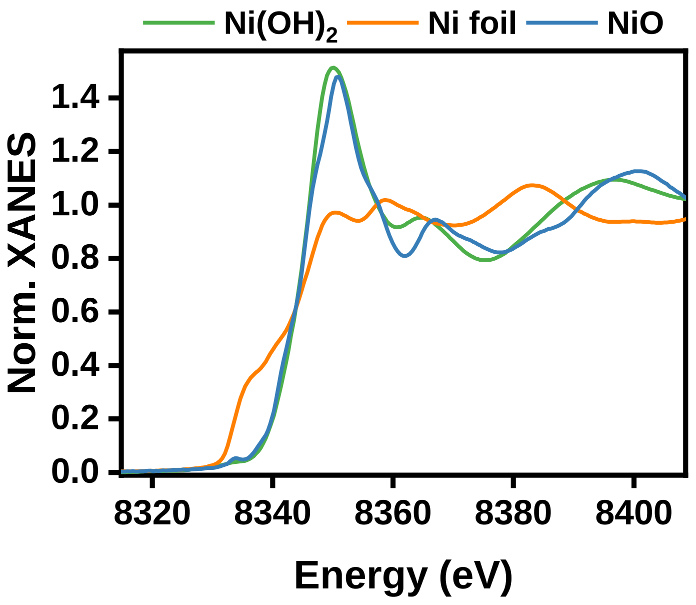

Figure S33. $Ni(OH)_2$, metallic Ni foil and NiO reference spectra.

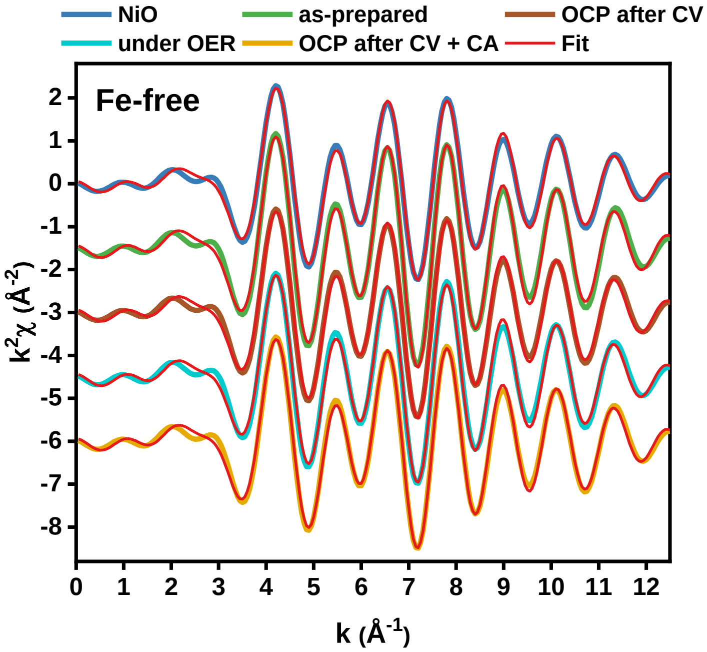

Figure S34. Fourier-filtered Ni K-edge EXAFS spectra in *k*-space and corresponding fits for measurements in 0.5 M borate buffer solution. The Fourier transform was carried out in the *k*-range from 3.0 $Å^{-1}$ up to 12.0 $Å^{-1}$.

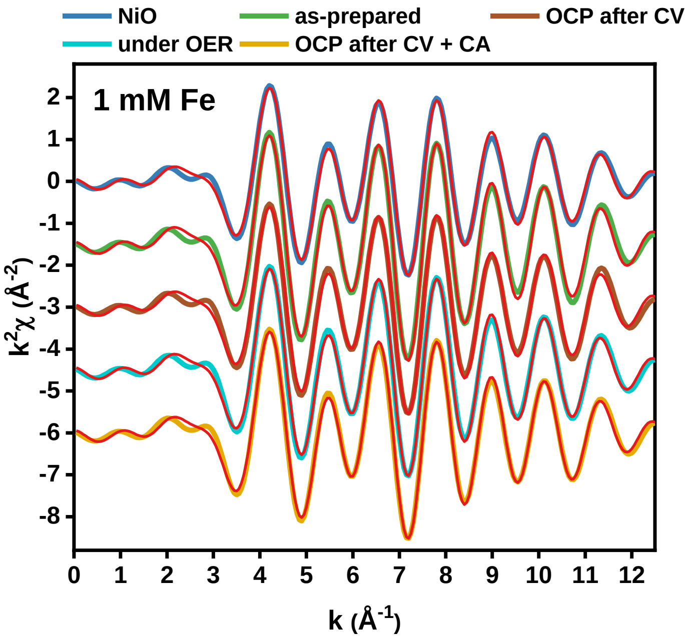

Figure S35. Fourier-filtered Ni K-edge EXAFS spectra in *k*-space and corresponding fits for measurements in 0.5 M borate buffer + 1 mM $Fe(NO_3)_3$ solution. The Fourier transform was carried out in the *k*-range from 3.0 $Å^{-1}$ up to 12.0 $Å^{-1}$.

Table S2. EXAFS fitting at the Ni K-edge with concurrent fitting of $\chi(k)k^1$, $\chi(k)k^2$ and $\chi(k)k^3$ in R-space in the range from 1.0-3.5 Å. Fitting was conducted for the first two coordination shells with two single scattering Ni-O and Ni-M paths. The parameters are the coordination number ***N***, the interatomic distance ***R***, the disorder factors $\boldsymbol{\sigma^2}$, the correction to the photoelectron reference energy $\Delta\boldsymbol{E_0}$. Fit quality parameter (***R factor***) is also given. The S_0^2 factor of 0.90 ± 0.04 was calculated by fixing the amplitude to N=6 for the Ni-O and N=12 for the Ni-M path.

#	Path	N	R (Å)	σ^2 (Å^2)	ΔE_0 (eV)	R factor
**NiO**	**Ni-O**	6	2.078 ± 0.004	0.0052 ± 0.0008	4.4 ± 0.5	1.2 %
	**Ni-M**	12	2.956 ± 0.002	0.0076 ± 0.0004		
**Dry**	**Ni-O**	5.7 ± 0.3	2.069 ± 0.004	0.0051 ± 0.0009	3.8 ± 0.3	0.9 %
	**Ni-M**	15.9 ± 0.8	2.954 ± 0.002	0.0079 ± 0.0004		
**OCP after CV (Fe-free)**	**Ni-O**	5.2 ± 0.3	2.075 ± 0.004	0.0050 ± 0.0007	3.5 ± 0.3	0.8 %
	**Ni-M**	14.4 ± 0.7	2.958 ± 0.002	0.0068 ± 0.0004		
**under OER (Fe-free)**	**Ni-O**	5.0 ± 0.2	2.075 ± 0.003	0.0045 ± 0.0007	3.5 ± 0.2	0.7 %
	**Ni-M**	15.5 ± 0.7	2.959 ± 0.002	0.0072 ± 0.0003		
**OCP after CV + CA (Fe-free)**	**Ni-O**	5.2 ± 0.2	2.074 ± 0.004	0.0047 ± 0.0007	3.6 ± 0.3	0.7 %
	**Ni-M**	14.7 ± 0.7	2.959 ± 0.002	0.0069 ± 0.0003		
**OCP after CV (1 mM Fe)**	**Ni-O**	5.3 ± 0.3	2.069 ± 0.004	0.0045 ± 0.0008	3.2 ± 0.3	1.0 %
	**Ni-M**	14.3 ± 0.8	2.955 ± 0.002	0.0066 ± 0.0004		
**under OER (1 mM Fe)**	**Ni-O**	5.5 ± 0.3	2.071 ± 0.004	0.0053 ± 0.0008	3.4 ± 0.3	0.8 %
	**Ni-M**	14.7 ± 0.7	2.957 ± 0.002	0.0068 ± 0.0004		
**OCP after CV + CA (1 mM Fe)**	**Ni-O**	5.6 ± 0.3	2.071 ± 0.004	0.0058 ± 0.0009	3.4 ± 0.3	0.9 %
	**Ni-M**	14.4 ± 0.8	2.956 ± 0.002	0.0067 ± 0.0004		

Table S3. Fe oxidation states and edge positions determined by integration of the absorption edge following Dau et al[17-18]. Error bars arise from the linear fit in Figure S29.

#	Oxidation State	Edge Position (eV)
**FeO**	2	7120.37
**Fe_3O_4**	2.67	7122.34
**α-Fe_2O_3**	3	7123.54
**FeOOH**	3	7123.71
**NiO_X + Fe + electrolyte**	3.05 ± 0.19	7123.75
**NiO_X + Fe + OCP after CV**	3.06 ± 0.19	7123.76
**NiO_X + Fe + under OER**	3.04 ± 0.19	7123.71
**NiO_X + Fe + OCP after CV + CA**	3.06 ± 0.19	7123.77

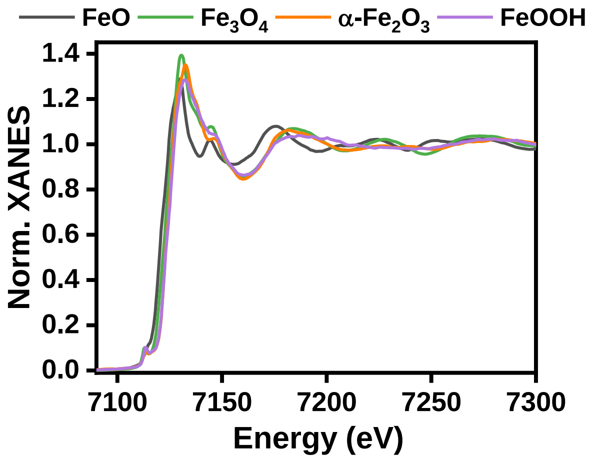

Figure S36. FeO, Fe_3O_4, α-Fe_2O_3 and FeOOH reference spectra.

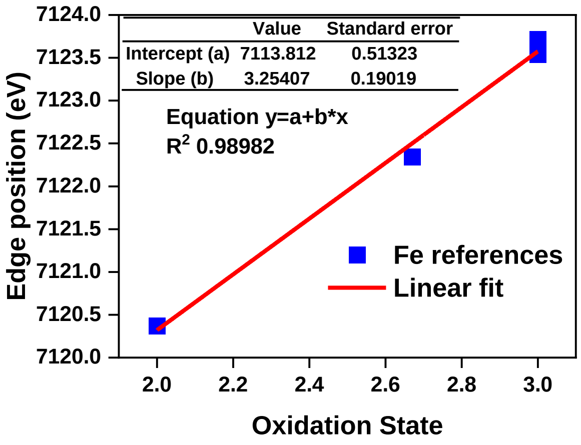

Figure S37. Linear correlation of absorption edge position and oxidation state for FeO_X reference compounds.

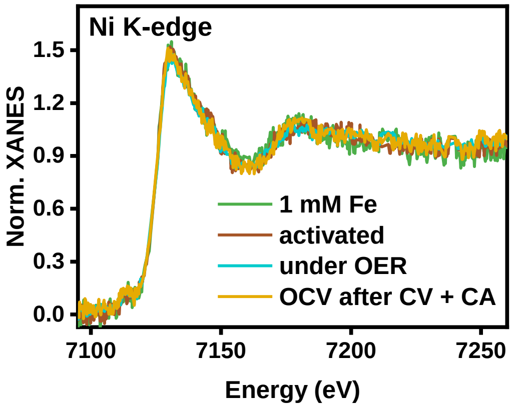

Figure S38. Normalized Fe K-edge XANES data for NiO_X catalyst in 0.5 M borate buffer + 1 mM $Fe(NO_3)_3$ solution.

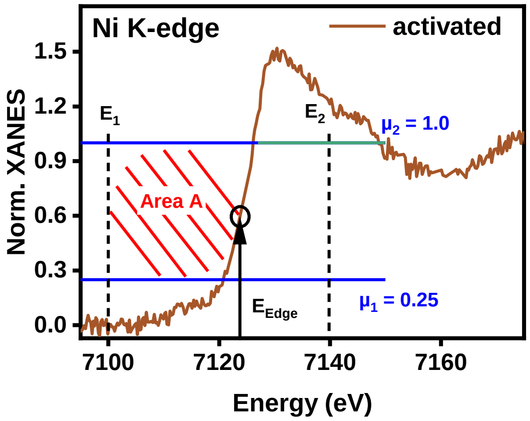

Figure S39. Schematic of the process using for the absorption edge integration, adapted from Dittmer et al.[18], following Dau et al.[17]. The integrated area of the absorption edge (blue) lies within the boundaries μ_1 and μ_2. Area A is enclosed by μ_1 and μ_2 in the y-axis and E_1 and $\mu X(E)$ in the x-axis. The edge position E_{Edge} results from $E_1 + A/(\mu_2 - \mu_1)$.

Thereby, the edge position E_{Edge} is calculated within an interval $[E_1, E_2]$:

$$E_{Edge} = E_1 + \frac{1}{\mu_2 + \mu_1} \int_{E_1}^{E_2} \mu_2 - \mu_X(E) dE$$

The function μ_0 is defined as:

$$\mu_X|(E) = \begin{cases} \mu_1 \; for \; \mu(E) < \mu_1 \\ \mu_2 \; for \; \mu(E) > \mu_2 \\ \mu(E) \; else \end{cases}$$

It should be noted that the edge position E_{Edge} does not depend on the interval borders [E_1 = 7100 eV, E_2 = 7140 eV] when using the boundaries μ_1 = 0.25 and μ_2 = 1 which were chosen to avoid impact of the pre-edge and the white-line[17].

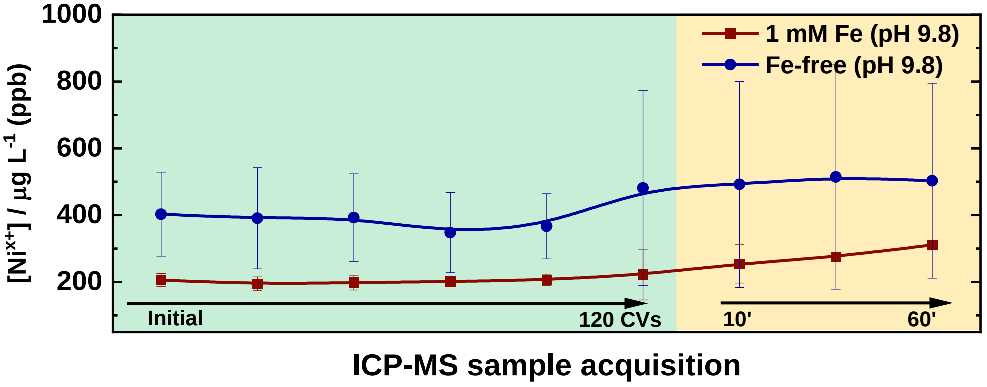

Figure S40. ICP-MS results of Ni element in in the electrolyte. Quantification of Fe uptake/loss in NiO octahedra was performed by replicating the EC-TEM experiments in an H-cell configuration. During H-cell experiments, a constant volume of 15 mL was employed per compartment: aliquots of 1 mL were withdrawn per each ICP-MS sample, followed by a 1mL injection of purified borate electrolyte to reinstate initial total volume. Besides the samples collected during cycling (0, 10, 20, 40, 60 and 120 CVs) and chronoamperometry (1mL every 10 mins) from the WE compartment, electrolyte was also collected after electrochemical testing from the CE compartment to monitor Fe crossover.

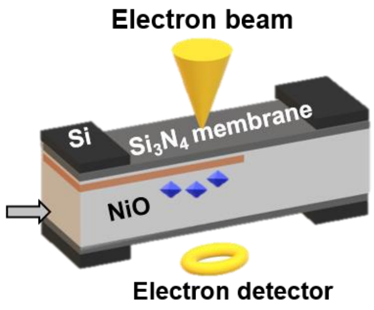

Figure S41. The schematic illustration of EC-TEM experiments performed with Si/SiN chips fitted with glassy carbon electrodes.

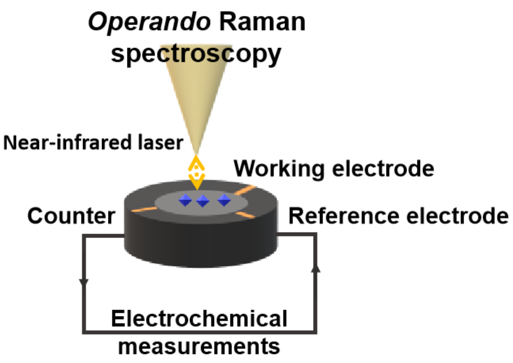

Figure S42. The schematic illustration of *operando* Raman experiments.

Supplementary Note 1: Control Experiments to Check for Electron-beam Induced Artifacts and Consistency to Standard Experimental Geometries

Here, we used the EC-TEM chips as working electrodes in a benchtop configuration to confirm the absence of artifacts due to the electron beam or EC-TEM cell geometry and to obtain more statistics regarding the Fe-induced changes. We also performed only CA experiments for durations up to 2 hours and the STEM-EDX results obtained at key time-points as determined from the *in situ* experiments are shown in Figure S41-43. In this case, we see increasing Fe deposition with time and extensive Fe deposition on both, the octahedra and the rest of the carbon working electrode support surface after 2 hours. We also compared the results obtained from samples reacted in 0.1 M KOH (pH 13) with samples reacted in the 0.5 M borate buffer (pH 9.8) to determine if similar changes took place in octahedra reacted in a conventional KOH electrolyte. Similar Fe-incorporated structures (Figure S44) were obtained in 1 mM Fe-containing KOH, indicating that the structural changes observed remain consistent in different electrolytes and setups. Comparative measurements with octahedra dropcasted on carbon paper (Figure S44) also confirmed that the results from experiments performed with the EC-TEM chips are consistent with samples extracted from benchtop measurements performed using conventional carbon supports.

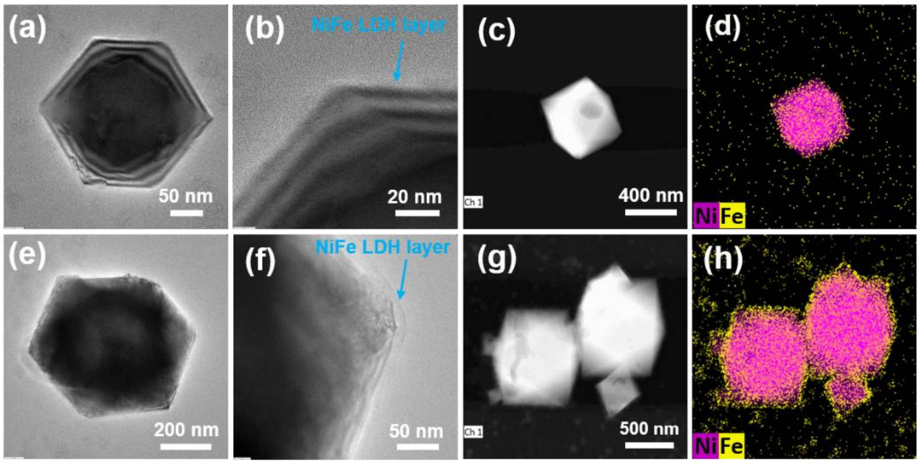

Figure S43. TEM images of NiO samples after chronoamperometric measurements for (a,b) 30 minutes and (e,f) 60 minutes at 1.7 V_{RHE} in 0.5 M borate buffer + 1 mM $Fe(NO_3)_3$ solution using a benchtop setup. EDX maps of NiO samples after chronoamperometric measurements for (c, d) 30 minutes and (g, h) 60 minutes at 1.7 V_{RHE} in a benchtop setup. The samples were dropcasted on a carbon electrode chip.

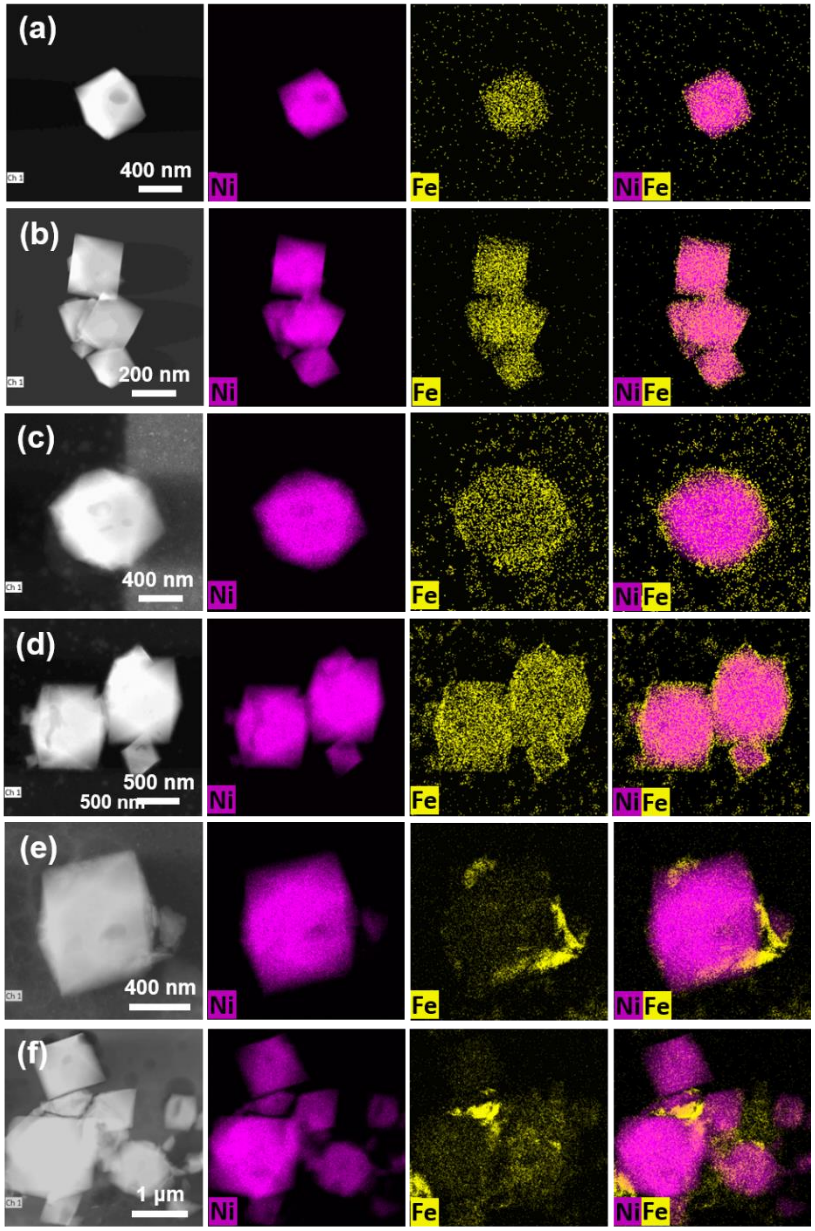

Figure S44. *Ex situ* EDX images of NiO after chronoamperometric measurements (a,b) for 30 minutes, (c,d) for 60 minutes, and (e,f) for 120 minutes at 1.7 V_{RHE} in 0.5 M borate buffer + 1 mM $Fe(NO_3)_3$ solution using a benchtop setup. The samples were dropcasted on a carbon electrode chip.

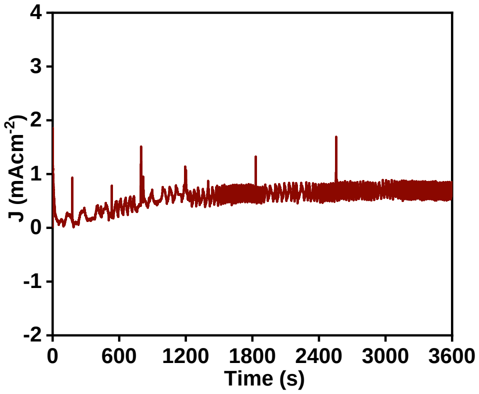

Figure S45. Chronoamperometric response for NiO without any CV cycles at 1.7 V_{RHE} in 0.5 M borate buffer + 1 mM $Fe(NO_3)_3$ solution using a benchtop setup. The sample was dropcasted on a carbon electrode chip.

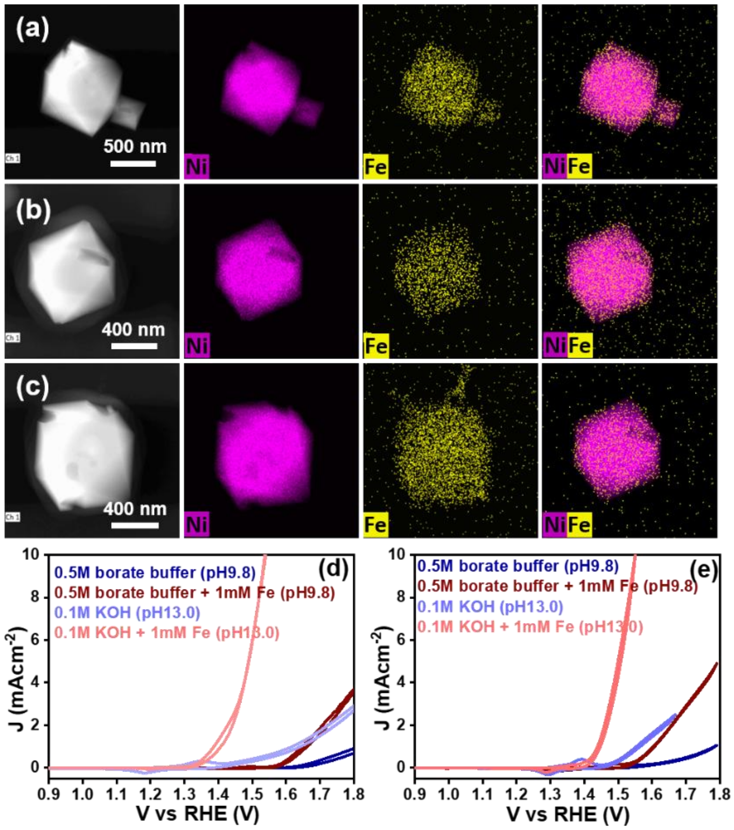

Figure S46. (a-c) *Ex situ* EDX images of NiO after chronoamperometric measurements for 30 minutes at 1.7 V_{RHE} in 0.1 M KOH +1 mM $Fe(NO_3)_3$ solution using a benchtop setup. (d,e) The comparison of cyclic voltammograms acquired with the NiO octahedra as electrocatalysts in 0.5 M borate buffer and 0.1 M KOH without/with Fe using our standard benchtop electrochemistry setup. Difference between the samples dropcasted on: (d) carbon paper, or (e) a carbon electrode chip. All potentials are IR corrected.

Supplementary Note 2: X-Ray Beam Parameters and Geometry for Fe Edge Measurements and a Discussion on Beam Effects and Measurement Instability due to Bubbles

At the P64 beamline, we used an X-ray beam that was not focused and had a beam size of 1 x 2 mm with 100 mA current. The synchrotron was operated at 6 GeV energy. In the *operando* XANES measurements, the signal of Fe K-edge XANES is damped by the comparably low, diluted concentration of Fe in the electrolyte and the geometry of incident X-ray beam with the cell, which include the electrodes, and the detector. The incident X-ray beam passes through the electrolyte and the emitted fluorescence is collected through the back of the sample, following Beer’s law. The detected fluorescence originates from Fe ions on the electrode and at the interface, hence, the Fe signal increases under the applied electrical field as more Fe ions get closer to the detector.

We can rule out beam effects in these measurements contributing to the enhanced Fe intensity measured during OER based on our measurements. Here, one possible scenario is that the X-ray beam causes the Fe deposition on the cell, which will contribute to a continuously increasing Fe edge intensity. However, as shown in Figure 5, Fe signal decreased when we repeated the measurement after OER and so, the edge intensity increase cannot be due to Fe deposition.

On the other hand, the Fe signal intensity varies during OER because of the pronounced formation of oxygen bubbles at the electrode. The formation and release of bubbles cause changes in the electrolyte background signal measured which result in stronger change in counts and a higher standard deviation. Nonetheless, the lowest intensity is still higher than those acquired under non-OER conditions.

Supplementary Note 3: Quantification of ICP-MS Results and Estimates of Bulk Ni to Fe Uptake Molar Ratios

Here, we can estimate the atomic ratio between Ni:Fe during the experiment from the mass of NiO octahedron dropcasted on the working electrode and change in the Fe uptake/loss from the ICP-MS measurements using the following calculations.

$$Bulk\ Ni{:}Fe\ ratio = \frac{m_{Ni,tot} - \Delta m_{Ni}}{\Delta m_{Fe}} * \frac{M_{Fe}}{M_{Ni}}$$

Where $m_{Ni,tot}$ is the total dropcasted Ni loading at the working electrodes, Δm_{Ni} the net mass uptake/loss of Ni estimated by ICP-MS, Δm_{Fe} the net mass uptake/loss of Fe estimated by ICP-MS, and lastly M_{Fe} and M_{Ni} the molecular weights of Fe and Ni, respectively.

Table S4 shows Δm_{Fe} is the net uptake/loss of Fe estimated by ICP-MS quantification and the subsequent conversion to bulk Ni : Fe atomic ratios.

Table S4. Average Fe mass uptake/loss and corresponding Ni : Fe bulk atomic ratios estimated from ICP-MS measurements after electrolyte withdrawal at different stages of the electrochemical protocol.

**Electrochemical step**	$\Delta\bar{m}_{Fe} \pm \sigma$ / µg	$Bulk\ Ni{:}Fe\ ratio(\pm\sigma)$
**Initial**	n.a.	n.a.
**10 CVs**	52 ± 2	1 : 1.02 (± 0.01)
**20 CVs**	86 ± 10	1 : 1.62 (± 0.07)
**40 CVs**	117 ± 29	1 : 2.2 (± 0.7)
**60 CVs**	141 ± 49	1 : 2.8 (± 1.1)
**120 CVs**	189 ± 70	1 : 3.8 (± 1.4)
**CA, 10 mins**	168 ± 50	1 : 3.4 (± 1)
**CA, 20 mins**	135 ± 13	1 : 2.8 (± 0.4)
**CA, 30 mins**	105 ± 2	1 : 2.2 (± 0.1)
**CA, 40 mins**	108.1 ± 0.1	1 : 2.3 (± 0.1)
**CA, 50 mins**	102 ± 22	1 : 2.2 (± 0.3)
**CA, 60 mins**	95 ± 20	1 : 2.1 (± 0.2)

File name: Supplementary Movie 1

Description: EC-TEM movie featuring the structural changes in the octahedral NiO catalysts during 17 cycles of cyclic voltammogram from 0.7 to 1.9 V_{RHE}. The NiO octahedra were dropcasted on an EC-TEM chip. It shows that there is no obvious change in the morphology of NiO with the applied potential. The plot on the right shows the electrochemical response as a function of the applied potential. The recording rate of the movie was 1 frame per second. The movie playback rate is in real time. The electron flux was 7 e^{-} $Å^{-2}$ s^{-1}, and the electron flux limit for observing noticeable beam-induced effects in our TEM is 56 e^{-} $Å^{-2}$ s^{-1}.

References

1. Ma, X.; Wang, N.; Qian, Y.; Bai, Z., Large-scale synthesis of NiO polyhedron nanocrystals as high-performance anode materials for lithium ion batteries. *Mater. Lett.* **2016,** *168*, 5-8.
2. Dincă, M.; Surendranath, Y.; Nocera, D. G., Nickel-borate oxygen-evolving catalyst that functions under benign conditions. *P. Natl. Aca. Sci.* **2010,** *107* (23), 10337-10341.
3. Risch, M.; Klingan, K.; Heidkamp, J.; Ehrenberg, D.; Chernev, P.; Zaharieva, I.; Dau, H., Nickel-oxido structure of a water-oxidizing catalyst film. *Chem. Commun.* **2011,** *47* (43), 11912-11914.
4. Trotochaud, L.; Young, S. L.; Ranney, J. K.; Boettcher, S. W., Nickel-iron oxyhydroxide oxygen-evolution electrocatalysts: the role of intentional and incidental iron incorporation. *J. Am. Chem. Soc.* **2014,** *136* (18), 6744-53.
5. Grosse, P.; Yoon, A.; Rettenmaier, C.; Herzog, A.; Chee, S. W.; Roldan Cuenya, B., Dynamic transformation of cubic copper catalysts during CO_2 electroreduction and its impact on catalytic selectivity. *Nat. Commun.* **2021,** *12* (1), 1-11.
6. Estrade-Szwarckopf, H., XPS photoemission in carbonaceous materials: A "defect" peak beside the graphitic asymmetric peak. *Carbon* **2004,** *42* (8-9), 1713-1721.
7. Ravel, B.; Newville, M., Data analysis for X-ray absorption spectroscopy using IFEFFIT. *J. Synchrotron Radiat.* **2005,** *12* (Pt 4), 537-541.
8. Newville, M., IFEFFIT: interactive XAFS analysis and FEFF fitting. *J. Synchrotron Radiat.* **2010,** *8* (Pt 2), 322-324.
9. Girod, R.; Nianias, N.; Tileli, V., Electrochemical behavior of carbon electrodes for in situ redox studies in a transmission electron microscope. *Micros. Microanal.* **2019,** *25* (6), 1304-1310.
10. Lu, Z.; Xu, W.; Zhu, W.; Yang, Q.; Lei, X.; Liu, J.; Li, Y.; Sun, X.; Duan, X., Three-dimensional NiFe layered double hydroxide film for high-efficiency oxygen evolution reaction. *Chem. Commun.* **2014,** *50* (49), 6479-6482.
11. El Mendili, Y.; Bardeau, J., Francois; Randrianantoandro, N.; Greneche, J., Marc; Grasset, F., Structural behavior of laser-irradiated γ-Fe_2O_3 nanocrystals dispersed in porous silica matrix: γ-Fe_2O_3 to α-Fe_2O_3 phase transition and formation of ε-Fe_2O_3. *Scie. Technol. Adv. Mate.* **2016,** *17* (1), 597-609.
12. El Mendili, Y.; Bardeau, J.-F.; Randrianantoandro, N.; Grasset, F.; Greneche, J.-M., Insights into the mechanism related to the phase transition from γ-Fe_2O_3 to α-Fe_2O_3 nanoparticles induced by thermal treatment and laser irradiation. *J. Phys. Chem. C.* **2012,** *116* (44), 23785-23792.
13. El Mendili, Y.; Bardeau, J. F.; Randrianantoandro, N.; Gourbil, A.; Greneche, J. M.; Mercier, A. M.; Grasset, F., New evidences of in situ laser irradiation effects on γ-Fe_2O_3 nanoparticles: a Raman spectroscopic study. *J. Raman Spectrosc.* **2011,** *42* (2), 239-242.
14. Lee, D. U.; Fu, J.; Park, M. G.; Liu, H.; Kashkooli, A. G. B. N.; Chen, Z., Self-Assembled NiO/$Ni(OH)_2$ Nanoflakes as Active Material for High-Power and High-Energy Hybrid Rechargeable Battery. *Nano Lett.* **2016,** *16*, 1794-1802.
15. Zhao, Z.; Liu, H.; Gao, W.; Xue, W.; Liu, Z.; Huang, J.; Pan, X.; Huang, Y., Surface-engineered PtNi-O nanostructure with record-high performance for electrocatalytic hydrogen evolution reaction. *J. Am. Chem. Soc.* **2018,** *140* (29), 9046-9050.
16. Lopez Luna, M.; Timoshenko, J.; Kordus, D.; Rettenmaier, C.; Chee, S. W.; Hoffman, A. S.; Bare, S. R.; Shaikhutdinov, S.; Roldan Cuenya, B., Role of the oxide support on the structural and chemical evolution of Fe catalysts during the hydrogenation of CO_2. *ACS Catal.* **2021,** *11* (10), 6175-6185.
17. Dau, H.; Liebisch, P.; Haumann, M., X-ray absorption spectroscopy to analyze nuclear geometry and electronic structure of biological metal centers--potential and questions examined with special focus on the tetra-nuclear manganese complex of oxygenic photosynthesis. *Anal. Bioanal. Chem.* **2003,** *376* (5), 562-583.
18. Dittmer, J.; Iuzzolino, L.; Dorner, W.; Nolting, H. F.; Meyer-Klaucke, W.; Dau, H., A new method for determination of the edge position of X-ray absorption spectra. *Photosynthesis: Mechanisms and Effects* **1998**, 1339-1342.
